# Efficacy of Niclosamide vs Placebo in SARS-CoV-2 Respiratory Viral Clearance, Viral Shedding, and Duration of Symptoms Among Patients With Mild to Moderate COVID-19: A Phase 2 Randomized Clinical Trial

**DOI:** 10.1001/jamanetworkopen.2021.44942

**Published:** 2022-02-09

**Authors:** Dana M. Cairns, Dorothy Dulko, Jeffrey K. Griffiths, Yoav Golan, Theodora Cohen, Ludovic Trinquart, Lori Lyn Price, Kirthana R. Beaulac, Harry P. Selker

**Affiliations:** 1 Department of Biomedical Engineering, Tufts University, Medford, Massachusetts; 2 Institute for Clinical Research and Health Policy Studies, Tufts Medical Center, Boston, Massachusetts; 3 Tufts Clinical and Translational Science Institute, Tufts University, Boston, Massachusetts; 4 Department of Public Health and Community Medicine, Tufts University School of Medicine, Boston, Massachusetts; 5 Division of Geographic Medicine and Infectious Diseases, Tufts Medical Center, Boston, Massachusetts; 6 Department of Pharmacy, Emerson Hospital, Concord, Massachusetts

## Abstract

**Importance:**

Oral anthelmintic niclosamide has potent in vitro antiviral activity against SARS-CoV-2. Repurposed niclosamide could be a safe and efficacious COVID-19 therapy.

**Objective:**

To investigate whether niclosamide decreased SARS-CoV-2 shedding and duration of symptoms among patients with mild to moderate COVID-19.

**Design, Setting, and Participants:**

This randomized, placebo-controlled clinical trial enrolled individuals testing positive for SARS-CoV-2 by polymerase chain reaction with mild to moderate symptoms of COVID. All trial participants, investigators, staff, and laboratory personnel were kept blind to participant assignments. Enrollment was among individuals reporting at Tufts Medical Center and Wellforce Network in Massachusetts for outpatient COVID-19 testing. The trial opened to accrual on October 1, 2020; the last participant enrolled on April 20, 2021. Trial exclusion criteria included hospitalization at time of enrollment or use of any experimental treatment for COVID-19, including vaccination. Enrollment was stopped before attaining the planned sample size when COVID-19 diagnoses decreased precipitously in Massachusetts. Data were analyzed from July through September 2021.

**Interventions:**

In addition to receiving current standard of care, participants were randomly assigned on a 1:1 basis to receive niclosamide 2 g by mouth daily for 7 days or identically labeled placebo at the same dosing schedule.

**Main Outcomes and Measures:**

Oropharyngeal and fecal samples were self-collected for viral shedding measured by reverse-transcriptase–polymerase-chain-reaction on days 3, 7, 10, and 14, and an additional fecal sample was collected on day 21. A telehealth platform was developed to conduct remote study visits, monitor symptoms, and coordinate sample collection via couriers. The primary end point was the proportion of participants with viral clearance in respiratory samples at day 3 based on the intention-to-treat sample. Mean times to viral clearance and symptom resolution were calculated as restricted mean survival times and accounted for censored observations.

**Results:**

Among 73 participants, 36 individuals were enrolled and randomized to niclosamide and 37 individuals to placebo. Participant characteristics were similar across treatment groups; among 34 patients receiving placebo and 33 patients receiving niclosamide in the intention-to-treat sample, mean (SD) age was 36.0 (13.3) years vs 36.8 (12.9) years and there were 21 (61.8%) men vs 20 (60.6%) men. The overall mean (SD) age was 36.4 (13.0) years. For the primary end point, 66.67% (95% CI, 50.74% to 81.81%) of participants receiving niclosamide and 55.88% (95% CI, 40.27% to 72.73%) of participants receiving placebo had oropharyngeal SARS-CoV-2 clearance at day 3 (*P* = .37). Among 63 participants with symptoms, niclosamide did not significantly shorten symptom duration, which was 12.01 (95% CI, 8.82 to 15.2) days in the niclosamide group vs 14.61 (95% CI, 11.25 to 17.96) days in the placebo group (mean difference, −2.6 [95% CI, −7.23 to 2.03] days). Niclosamide was well-tolerated; the most commonly reported adverse events in the placebo and niclosamide groups were headaches (11 patients [32.4%] vs 7 patients [21.2%]; *P* = .31) and cough (8 patients [23.5%] vs 7 patients [21.2%]; *P* = .82).

**Conclusions and Relevance:**

In this randomized clinical trial, there was no significant difference in oropharyngeal clearance of SARS-CoV-2 at day 3 between placebo and niclosamide groups. Confirmation in larger studies is warranted.

**Trial Registration:**

ClinicalTrials.gov Identifier: NCT04399356

## Key Points

**Question:**

Does oral niclosamide decrease the contagious period as determined by SARS-CoV-2 shedding among patients with mild to moderate COVID-19?

**Findings:**

In this randomized clinical trial that included 73 adults with mild to moderate COVID-19, the proportion of participants achieving oropharyngeal clearance of SARS-CoV-2 at 3 days postenrollment was not statistically significantly different between patients given placebo and those given niclosamide. Niclosamide was well-tolerated.

**Meaning:**

This study did not find a significant effect of niclosamide on decreasing the contagious period of SAR-CoV-2 infection.

## Teaser

This randomized clinical trial compares the efficacy of niclosamide vs placebo in respiratory clearance of SARS-CoV-2 among patients with mild to moderate COVID-19.

## Introduction

There are no currently available effective Food and Drug Administration–approved oral treatments for COVID-19. Although novel therapeutics are being developed, repurposing known safe and accessible medications with promise for COVID-19 could facilitate more rapid introduction into clinical practice. Niclosamide, an anthelmintic discovered in 1958,^1^ works by blocking sugar uptake by the parasitic worm. It is on the World Health Organization (WHO) Essential Medicines List, which includes the safest and most effective medicines needed in health systems. The wholesale cost of this drug is approximately $0.24 USD for a course of treatment.^1^

Shortly after the SARS outbreak in China, niclosamide was found to inhibit SARS coronavirus, SARS-CoV, in vitro^2^ and in vivo.^3^ More recently, niclosamide was shown to have antiviral activity against SARS-CoV-2, the strain responsible for the COVID-19 pandemic, demonstrating 40-fold increased potency compared with remdesivir in vitro.^4,5^

There are several proposed antiviral mechanisms of action of niclosamide against COVID-19. Niclosamide has been reported to neutralize endolysosomal pH, preventing cell entry of pH-dependent viruses, such as SARS-CoV-2.^6^ Niclosamide also has been suggested to inhibit RNA viruses at a postentry stage, during viral RNA replication.^7^ More recently, niclosamide was shown to prevent viral replication via the inhibition of SARS-CoV-2 spike protein–mediated cell fusion.^8^ In addition to its anthelmintic and antiviral properties, niclosamide has demonstrated anti-inflammatory activity^9,10^ and promise for respiratory illness,^2,11,12^ including bronchodilator activity in a mouse model of asthma.^11,12^ Niclosamide has been repurposed for use in multiple clinical trials for cancer therapy^13,14,15^ and is tolerated in patients who have cancer and are immunocompromised.

Although absorbed systemically, niclosamide is concentrated in the gastrointestinal tract. Nearly 30% of patients with COVID-19 present gastrointestinal symptoms alone,^16^ highlighting the potential importance of the intestine in SARS-CoV-2 pathophysiology. In a 2020 study^16^ evaluating clinical samples from 74 hospitalized patients with COVID-19, there were 39 individuals with stool samples testing positive for SARS-CoV-2 RNA. Fecal viral shedding continued as long as 5 weeks after the last detection of SARS-CoV-2 RNA in respiratory samples, suggesting that the gastrointestinal tract may serve as a viral reservoir and allow for prolonged COVID-19 infection and potentially transmission.^17^

Prevention of COVID-19 by vaccines is crucial, but active, as well as passive immunity are limited by the emergence of new SARS-CoV-2 variants. Given the reported antiviral mechanism of action of niclosamide against SARS-CoV-2 in vitro,^8,18,19^ we hypothesized that niclosamide would prevent viral replication in the clinically relevant outcome of viral shedding from respiratory and intestinal routes and that this decrease in viral burden could attenuate symptomatic COVID-19. We conducted a phase 2 randomized, double-blind, placebo-controlled clinical trial to study the effects of niclosamide on viral clearance and duration of symptoms among patients with mild to moderate COVID-19.

## Methods

**Figure 1.**
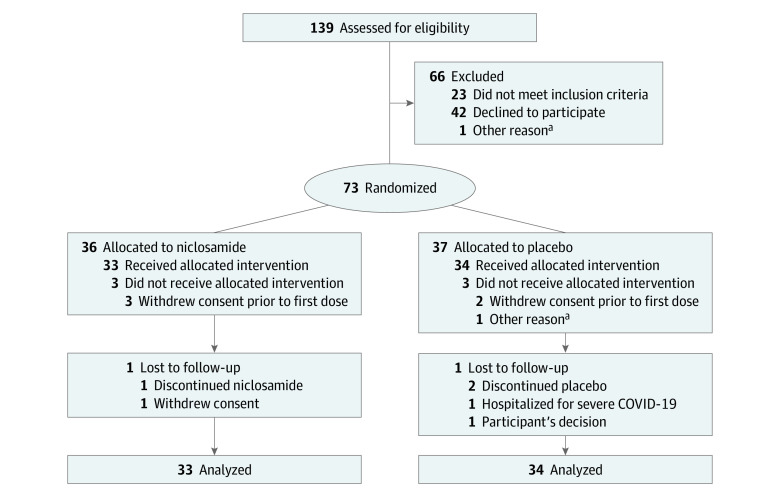
Flow Diagram The flow diagram summarizes the number of participants who were assessed for eligibility at screening, eligible at screening, eligible and randomized, withdrawn prior to receiving first dose, and randomized and included in the primary analysis. The flow diagram also depicts those who were eligible but not randomized, did not receive the randomized allocation, were lost to follow-up, and discontinued the intervention. ^a^Participant cohabited with a previously enrolled participant.

This randomized placebo-controlled clinical trial was approved by the Tufts Medical Center and Tufts University Human Investigations Committee. The study is registered at ClinicalTrials.gov (NCT04399356). Informed consent was obtained by physician investigators (J.K.G. and Y.G.) via a telehealth platform. This report follows the Consolidated Standards of Reporting Trials (CONSORT) reporting guideline (Figure 1).

### Inclusion and Exclusion Criteria

This study enrolled individuals testing positive for SARS-CoV-2 by polymerase chain reaction (PCR) who were asymptomatic or had mild to moderate symptoms of COVID disease. Enrollment was among individuals reporting at Tufts Medical Center and Wellforce Network in Massachusetts for outpatient COVID-19 testing. The trial opened to accrual on October 1, 2020; the last participant enrolled on April 20, 2021. Trial exclusion criteria included need for supplemental oxygen or requirement for hospitalization at the time of enrollment, participation in another related trial, or use of any experimental treatment for COVID-19 (including vaccination). Enrollment was stopped before attaining the planned sample size when COVID-19 diagnoses decreased precipitously in Massachusetts. Information in eMethods in Supplement 1 details study methods and specific inclusion and exclusion criteria. The trial protocol is included in Supplement 2.

### Randomization and Intervention

In addition to receiving current standard of care, participants were randomly assigned on a 1:1 basis to receive niclosamide 2 g by mouth daily for 7 days (ie, the experimental group) or placebo using the same dosing schedule (ie, the control group). Bayer Pharmaceuticals provided niclosamide. Tablets of niclosamide and placebo were similar in appearance and were dispensed in blinded blister packages labeled in accordance with state and federal regulations. All trial participants, investigators, staff, and laboratory personnel were kept blind to participant assignments. Independent, unblinded pharmacists from Tufts Investigational Drug Services dispensed niclosamide or placebo. A study kit, including pills, pill diary, thermometer, study diary, and pulse oximeter, was delivered via courier to all participants upon enrollment. All pills were dispensed at one time.

### Primary and Secondary Outcomes

The a priori primary efficacy end point was the proportion of participants with viral clearance in respiratory samples at day 3 based on the intention-to-treat (ITT) sample. Secondary end points included proportion of participants with viral clearance in fecal samples at day 14, change in respiratory viral shedding (on days 1, 3, 7, 10, and 14), progression to severe COVID disease, and time to resolution of symptoms reported on day 1. The safety end point was defined as the incidence of any adverse event. Identification and analysis based on fecal shedding status were performed as post hoc analyses.

### Telehealth Platform for Study Visits

A Health Insurance Portability and Accountability Act (HIPAA)–compliant telehealth platform was used to conduct all study visits remotely. This platform was also used to monitor participant self-report of symptoms and adverse events at days 1 through 7, 10, 14, 21, and 30 and to obtain self-reported race and ethnicity. Reporting race and ethnicity in this study was mandated by the US National Institutes of Health (NIH), consistent with the Inclusion of Women, Minorities, and Children policy.

### Oropharyngeal and Fecal Sample Collection and Processing

Oropharyngeal and fecal swabs were collected at days 1, 3, 7, 10, and 14, and an additional fecal sample was collected on day 21. Samples were self-collected by participants to avoid unnecessary hospital visits and to encourage participant compliance with self-quarantine. Supervised self-collected oral fluid and anterior nasal swab specimens have been found to perform similarly to clinician-collected nasopharyngeal swab specimens for the detection of SARS-CoV-2.^20^ Collection of oropharyngeal samples was observed via the telehealth platform by a study team member (D.D., J.K.G., and Y.G.) to ensure that sampling methods were consistent for each participant across all times. The samples were returned to a Clinical Laboratory Improvement Amendments–certified laboratory via FedEx. Samples were assayed for the presence or absence of SARS-CoV-2 viral RNA using quantitative real-time PCR. Viral loads were calculated based on cycle threshold (Ct) values from real time amplification.

### Statistical Analysis

The trial was powered for the primary end point. We estimated that including 40 participants in each group would achieve 89% power, with a 2-sided α level of .05, to detect a between-group difference in oropharyngeal viral clearance at day 3 of 35%, assuming that 15% of participants in the placebo group and 50% of participants in the niclosamide group would have cleared on day 3. We defined viral clearance as the first day a participant’s sample result was negative, provided that no subsequent sample results were positive. We estimated the cumulative probability of clearance in randomization groups using the Kaplan-Meier estimator. The restricted mean time to clearance was defined as the area under the viral clearance–free survival curve. This takes censored observations into account and is not based only on participants who achieved clearance.

For the primary end point of oropharyngeal viral clearance and the secondary end point of fecal viral clearance, we compared clearance probabilities at day 3 and day 14 between groups using χ^2^ tests based on the log of −log transformation for the survival function.^21^ We also compared restricted mean times to viral clearance up to day 14 for oropharyngeal viral clearance and day 21 for fecal viral clearance.^22,23^

We evaluated time to symptom resolution for participants who reported at least 1 ongoing or new symptom at day 1. Symptom resolution was defined as the first day that all symptoms reported at day 1 had resolved. We compared restricted mean survival times to symptom resolution up to day 30.

In prespecified subgroup analyses, we compared viral clearance and time to symptom resolution between niclosamide and placebo groups among participants with body mass index (BMI; calculated as weight in kilograms divided by height in meters squared) scores in the reference range (ie, BMI < 25) and overweight scores (ie, BMI ≥ 25). In post-hoc subgroup analyses, we compared viral clearance and time to symptom resolution between niclosamide and placebo groups among participants with and without fecal shedding. All participants had a positive PCR test by oropharyngeal sample as part of the inclusion criteria. We defined individuals with double shedding as participants who also had at least 1 positive fecal sample at any time during the trial.

Analyses used SAS Studio statistical software version 3.8 (SAS Institute) and R statistical software version 4.1.0 (R Project for Statistical Computing). All statistical tests were 2-sided using a 5% significance level, and 95% CIs were 2-sided. A complete statistical analysis plan is included in Supplement 2. Data were analyzed from July through September 2021.

## Results

### Participant Characteristics

Among 73 participants, 36 individuals were enrolled and randomized to niclosamide and 37 individuals to placebo (Figure 1); 6 participants (3 in each group) withdrew consent before day 1 and before taking the first pill. All other participants returned at least 1 oropharyngeal sample, but 2 participants in the placebo group did not return fecal samples. Thus, ITT analyses based on oropharyngeal samples included 67 participants (33 individuals in the niclosamide group and 34 individuals in the placebo group), and ITT analyses based on fecal samples included 65 participants (33 individuals in the niclosamide group and 32 individuals in the placebo group). Participant characteristics were similar in placebo vs niclosamide treatment groups; in the intention-to-treat group with oropharyngeal samples, mean (SD) age was 36.0 (13.3) years vs 36.8 (12.9) years and there were 21 (61.8%) men vs 20 (60.6%) men (Table 1). The overall mean (SD) age was 36.4 (13.0) years. Among all patients in the ITT sample, there were 4 African American individuals (6.0%), 5 Asian individuals (7.5%), 7 Hispanic individuals (10.4%), 53 White individuals (79.1%), 1 individual with multiracial or multiethnic background (self-reported Asian and White; 1.5%), and 4 individuals with other race or ethnicity (6.0%). The other race and ethnicity category included individuals with Middle Eastern backgrounds or undisclosed race or ethnicity.

**Table 1.** Baseline and Demographic Characteristics of Participants

Characteristic	Participants, No. (%)		
	All patients (N = 67)	Placebo (N = 34)	Niclosamide (N = 33)
Age, mean (SD), y	36.39 (13.01)	35.97 (13.27)	36.82 (12.92)
Sex			
Men	41 (61.2)	21 (61.8)	20 (60.6)
Women	26 (38.8)	13 (38.2)	13 (39.4)
Race and ethnicity			
African American or Black	4 (6.0)	3 (8.8)	1 (3.0)
Asian	5 (7.5)	2 (5.9)	3 (9.1)
Hispanic or Latinx	7 (10.4)	1 (2.9)	6 (18.2)
White	53 (79.1)	27 (79.4)	26 (78.8)
Multiracial or multiethnic^a^	1 (1.5)	1 (2.9)	0
Other^b^	4 (6.0)	1 (2.9)	3 (9.1)
Smoking status			
Nonsmoker	51 (76.1)	27 (79.4)	24 (72.7)
Former smoker	10 (14.9)	4 (11.8)	6 (18.2)
Current smoker	6 (9.0)	3 (8.8)	3 (9.1)
Medical condition			
Heart condition	1 (1.5)	1 (2.9)	0
Asthma	5 (7.5)	4 (11.8)	1 (3.0)
Hypertension	5 (7.5)	2 (5.9)	3 (9.1)
BMI^c^			
Reference range	26 (45.6)	13 (46.4)	13 (44.8)
Overweight	24 (42.1)	10 (35.7)	14 (48.3)
Obese	4 (7.0)	4 (14.3)	0
Severely obese	3 (5.3)	1 (3.6)	2 (6.9)
Mean (SD)	27.04 (6.73)	27.25 (7.91)	26.83 (5.49)
Pills used, median (IQR), No.	28.00 (28.00-28.00)	28.00 (28.00-28.00)	28.00 (28.00-28.00)

Abbreviation: BMI, body mass index (calculated as weight in kilograms divided by height in meters squared).

^a^ One participant self-reported as Asian and White.

^b^ The other race and ethnicity category includes individuals with Middle Eastern backgrounds or undisclosed race and ethnicity.

^c^ BMI categories were reference range (<25), overweight (25 to <30), obese (30 to <35), and severely obese (≥35).

### Clearance of SARS-CoV-2 From Oropharyngeal Samples

The a priori primary outcome, oropharyngeal clearance at day 3, was 66.67% (95% CI, 50.74% to 81.81%) in the niclosamide group and 55.88% (95% CI, 40.27% to 72.73%) in the placebo group (*P* = .37) (Table 2). For the primary outcome of respiratory clearance of SARS-CoV-2, there was no significant difference in the restricted mean time to clearance between groups, at 3.39 (95% CI, 1.88 to 4.91) days with niclosamide and 3.44 (95% CI, 2.23 to 4.65) days with placebo, for a mean difference of −0.05 (95% CI, −1.99 to 1.90) days. Based on SARS-CoV-2–specific nucleocapsid N1 expression (normalized using RNase P values), there were no significant differences in mean capsid expression between treatment groups at days 1, 3, 7, 10, or 14.

**Table 2.** Oropharyngeal Clearance of SARS-CoV-2 at Day 3

Treatment group	Oropharyngeal SARS-CoV2 clearance at day 3, % (95% CI)
Placebo (n = 34)	55.88 (40.27-72.73)
Niclosamide (n = 33)	66.67 (50.74-81.81)

### Participants Who Shed SARS-CoV-2 in Oropharyngeal and Fecal Samples

**Figure 2.**
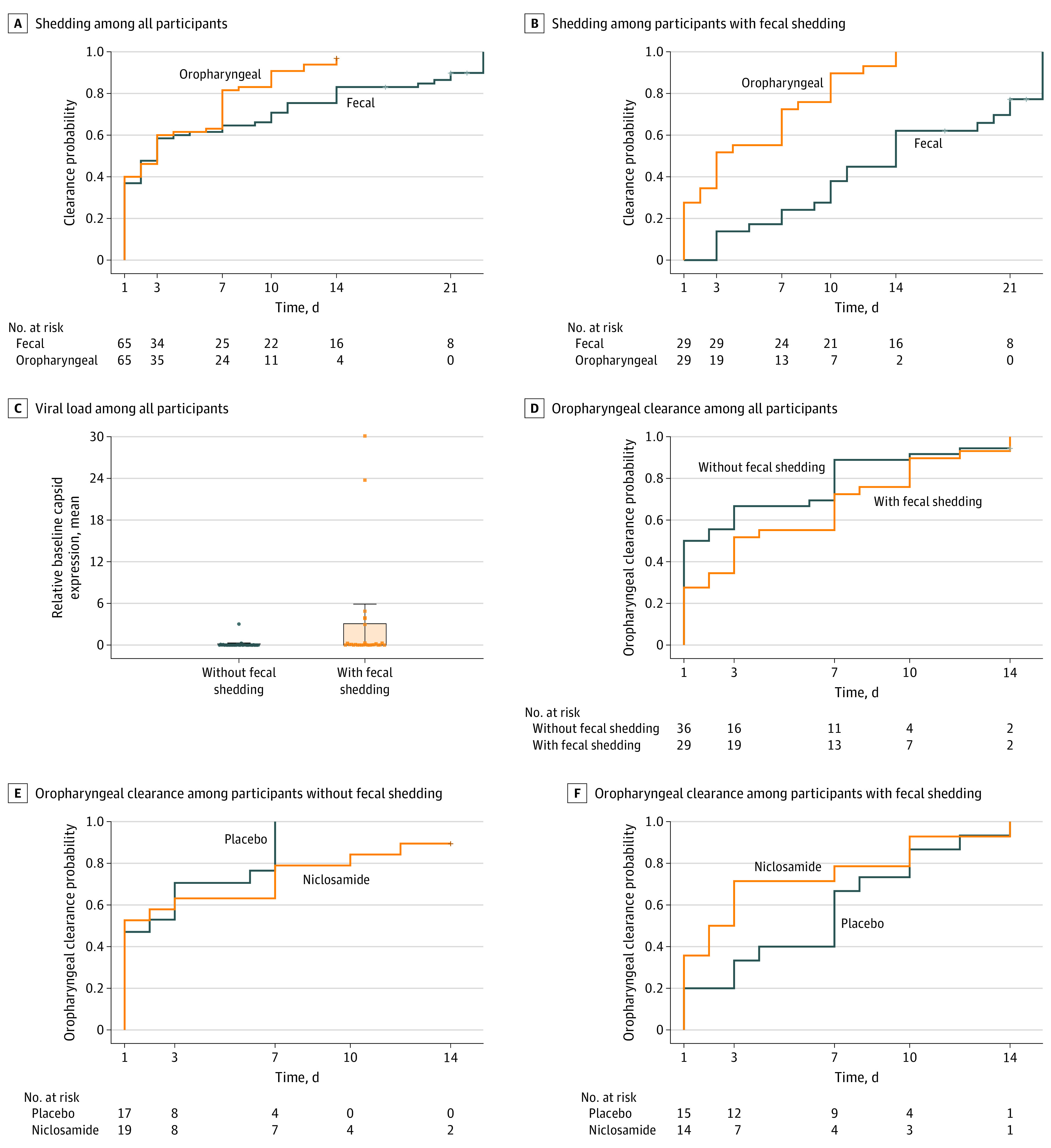
SARS-CoV-2 Shedding Among Participants With and Without Fecal Shedding A-B, Oropharyngeal and fecal shedding of SARS-CoV-2 is presented among all 65 participants and 29 participants with fecal shedding. C, Normalized baseline capsid expression (points) and mean viral load (shaded areas) among all 65 participants, including those without fecal shedding and those with fecal shedding, based on expression of SARS-CoV-2–specific nucleocapsid N1 (normalized using *RNase P* values) on day 1. Whiskers indicate 95% CIs. D, Oropharyngeal clearance of SARS-CoV-2 is presented among all 65 patients, including those without and those with fecal shedding. E-F, Oropharyngeal clearance of SARS-CoV-2 is presented by treatment group among 36 patients without and 29 patients with fecal shedding.

Detectable levels of SARS-CoV-2 were found in oropharyngeal and fecal samples. The timelines of oropharyngeal and fecal shedding by treatment group are compared graphically in eFigure 1 in Supplement 1. SARS-CoV-2 persisted longer in stool than respiratory samples (Figure 2A). The mean time to viral clearance up to day 14 was 3.51 (95% CI, 2.52 to 4.5) days in oropharyngeal samples and 4.82 (95% CI, 3.49 to 6.14) days in fecal samples, for a mean difference of −1.31 (95% CI, −2.96 to 0.35) days, which was not statistically significant.

All participants had a positive PCR test by oropharyngeal sample as part of inclusion criteria. In post-hoc analyses, we defined patients with double shedding as participants who also had at least 1 positive fecal sample during the study. Of 65 participants with at least 1 fecal sample, 29 individuals (44.6%) had fecal shedding and 36 individuals (55.4%) had no fecal shedding (Figure 2B). Among patients with fecal shedding, mean time to viral clearance up to day 14 was 4.31 (95% CI, 2.79 to 5.83) days based on oropharyngeal samples and 9.9 (95% CI, 8.42 to 11.37) days based on fecal samples, for a mean difference of −5.59 (95% CI, −7.70 to −3.47) days. In addition, patients with fecal shedding had an increased mean viral load compared with patients without fecal shedding based on SARS-CoV-2–specific nucleocapsid N1 baseline expression (normalized using RNase P values) on day 1 (*P* = .01) (Figure 2C).

Patients without fecal shedding had a decreased viral load (Figure 2C) and cleared SARS-CoV-2 from oropharyngeal samples faster compared with patients with fecal shedding (Figure 2D). The mean time to oropharyngeal clearance was 4.31 (95% CI, 2.79 to 5.83) days among patients with fecal shedding and 2.86 (95% CI, 1.60 to 4.13) days among patients without fecal shedding, for a mean difference of 1.45 (95% CI, −0.53 to 3.43) days, which was not statistically significant.

### Clearance of SARS-CoV-2 From Oropharyngeal Samples by Fecal Shedding Status

Among 36 patients without fecal shedding, the mean time to clearance was 3.53 (95% CI, 1.42 to 5.63) days in the niclosamide group and 2.12 (95% CI, 0.93 to 3.3) days in the placebo group, for a mean difference of 1.41 (95% CI, −1.01 to 3.83) days, which was not statistically significant (Figure 2E). Among 29 patients with fecal shedding, the mean time to viral clearance based on oropharyngeal samples was not significantly different between treatment groups (Figure 2F). The mean time to oropharyngeal clearance up to day 14 was 3.21 (95% CI, 1.06 to 5.37) days with niclosamide vs 5.33 (95% CI, 3.33 to 7.34) days with placebo, for a mean difference of −2.12 (95% CI, −5.06 to 0.82) days.

### Clearance of SARS-CoV-2 From Fecal Samples

Across all participants in our study, the mean time to fecal SARS-CoV-2 clearance was 6.12 (95% CI, 3.51 to 8.73) days in the niclosamide group and 5.77 (95% CI, 3.3 to 8.23) days in the placebo group (eFigure 2 in Supplement 1). For all participants, the mean difference was 0.36 (95% CI, −3.23 to 3.95) days, which was not statistically significant. Among patients with fecal shedding, mean time to fecal viral clearance in the niclosamide group was 13.21 (95% CI, 9.75 to 16.68) days vs 11.70 (95% CI, 8.47 to 14.93) days in the placebo group (eFigure 3 in Supplement 1). For these patients, the mean difference was 1.51 (95% CI, −3.22 to 6.25) days, which was not statistically significant.

### Progression to Severe Disease and Time to Resolution of COVID-19–Related Symptoms

**Figure 3.**
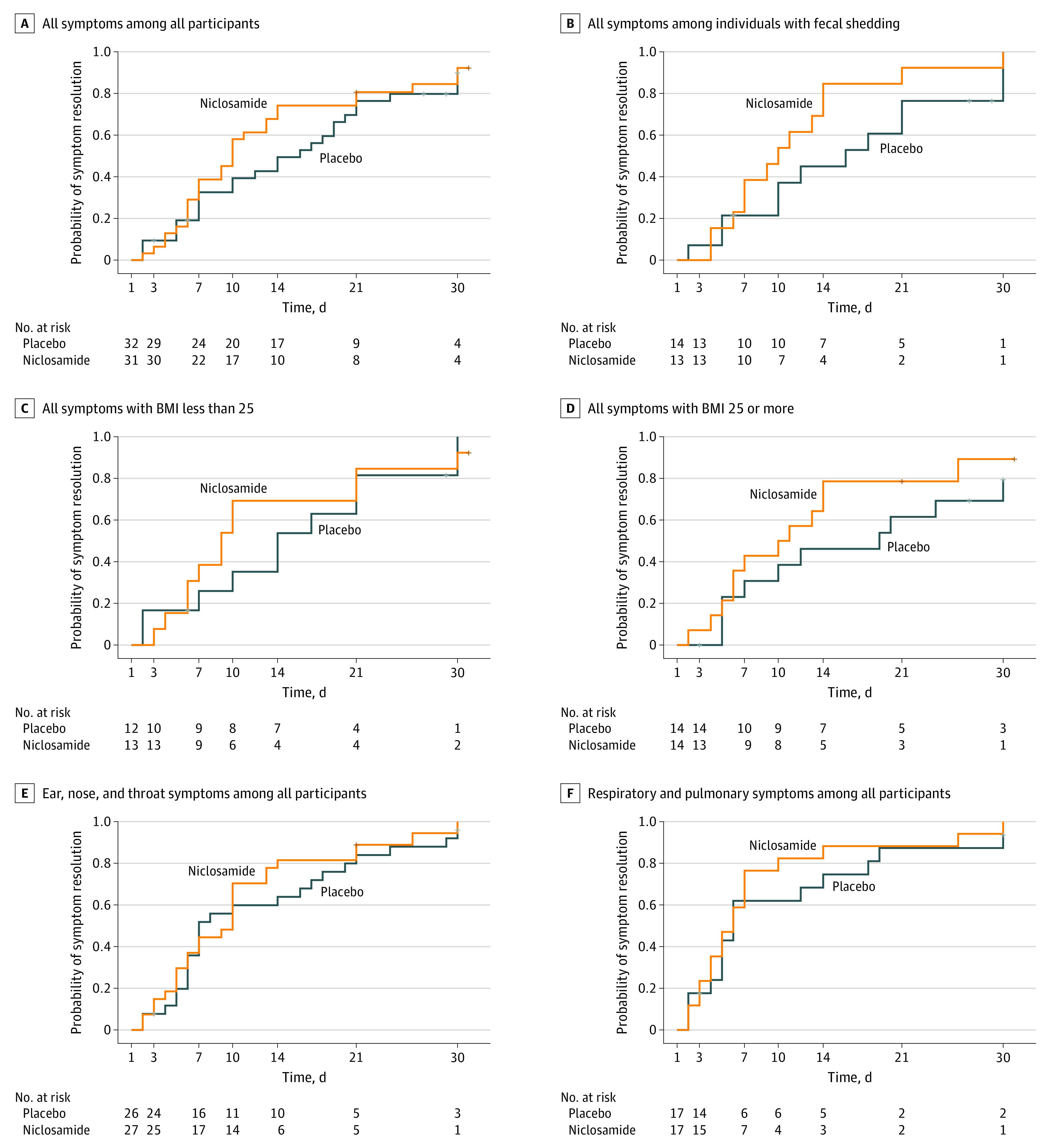
Mean Time to Symptom Resolution Among Patients With COVID-19 A-D, Mean time to all symptom resolution is presented among patients with COVID-19 for all 63 participants, 27 patients with fecal shedding, 25 patients with body mass index (BMI) scores less than 25, and 28 patients with BMI scores of 25 or greater. E-F, Mean time to resolution of oropharyngeal and respiratory symptoms related to COVID-19 is presented among all participants.

There was 1 participant (2.9%) in the placebo group and there were no participants in the niclosamide group who progressed to severe COVID-19 disease, while 2 patients without symptoms enrolled in our study because they tested positive after known exposure to COVID-19. Time to resolution of all symptoms in the primary cohort of 63 participants with symptoms was 12.01 (95% CI, 8.82 to 15.2) days in the niclosamide group vs 14.61 (95% CI, 11.25 to 17.96) days in the placebo group, for a mean difference of −2.6 (95% CI, −7.23 to 2.03) days, which was not statistically significant (Figure 3A). Among 27 patients with fecal shedding and increased viral load, the mean time to symptom resolution was 10.54 (95% CI, 6.73 to 14.35) days with niclosamide vs 15.41 (95% CI, 10.36 to 20.47) days with placebo, for a mean difference of −4.88 (95% CI, −11.21 to 1.45) days, which was not statistically significant (Figure 3B).

Some participants were in known high-risk groups for progression to severe COVID-19, including 28 participants with symptoms who were considered to have overweight or obesity (ie, BMI ≥ 25). The mean time to symptom resolution among these participants was 11.57 (95% CI, 6.92 to 16.23) days with niclosamide vs 16.46 (95% CI, 10.94 to 21.98) days with placebo, for a mean difference of −4.89 (95% CI, −12.11 to 2.33) days, which was not statistically significant (Figure 3D).

For COVID-related symptoms affecting the ear, nose, and throat (ENT; ie, sore throat, congestion, or loss of taste or smell), the mean time to symptom resolution was 9.74 (95% CI, 6.8 to 12.68) days with niclosamide vs 11.31 (95% CI, 7.87 to 14.75) days with placebo, for a mean difference of −1.57 (95% CI, −6.1 to 2.95) days, which was not statistically significant (Figure 3E). For COVID-19–related symptoms affecting respiratory or pulmonary tissues (ie, cough, dyspnea, or hypoxia), the mean time to symptom resolution was 7.29 (95% CI, 3.59 to 10.99) days with niclosamide vs 9.49 (95% CI, 5.04 to 13.93) days with placebo, for a mean difference of −2.19 (95% CI, −7.98 to 3.59) days, which was not statistically significant (Figure 3F). There was no difference between treatment groups in non-ENT, nonrespiratory symptoms, such as those affecting the central nervous system (eFigure 4 in Supplement 1), those affecting musculoskeletal tissues (eFigure 5 in Supplement 1), or general fever or chills (eFigure 6 in Supplement 1).

Niclosamide was well-tolerated (eTable in Supplement 1). In the ITT sample, the most commonly reported adverse events in the placebo and niclosamide groups were headaches (11 patients [32.4%] vs 7 patients [21.2%]; *P* = .31) and cough (8 patients [23.5%] vs 7 patients [21.2%]; *P* = .82).

## Discussion

In this randomized clinical trial’s primary end point, 66.7% of participants in the niclosamide group had oropharyngeal SARS-CoV-2 clearance at day 3 vs 55.9% of participants in the placebo group (*P* = .37). That this end point was not significantly different between groups may be explained by the large percentage of participants with relatively low viral loads who cleared virus from respiratory tissues by day 3, regardless of treatment group.

Tests for SARS-CoV-2 in oropharyngeal and fecal samples showed that the virus persists longer in the feces, as previously reported.^17^ In post-hoc analysis, we identified a subset of 29 of 65 participants who shed SARS-CoV-2 in oropharyngeal and fecal samples, designated as having double shedding. At baseline, individuals with fecal shedding had increased viral loads compared with participants without fecal shedding, designated as having single shedding. Individuals with fecal shedding took longer to achieve oropharyngeal clearance than those without fecal shedding. Increased viral load has been associated with increased level of disease.^24^ It is plausible that participants with fecal shedding had increased overall viral loads, which may be related to their increased disease severity compared with individuals without fecal shedding. Niclosamide had a minimal effect among patients without fecal shedding, who cleared SARS-CoV-2 relatively quickly. There was a decreased time to oropharyngeal clearance among patients with fecal shedding and a shorter time to resolution of symptoms with niclosamide compared with placebo overall (12.01 days vs 14.61 days), with a greater difference among patients with fecal shedding (10.54 days vs 15.41 days); however, these differences were not statistically significant. The niclosamide-associated decrease in symptoms was related to oropharyngeal clearance of SARS-CoV-2 among patients with ENT or respiratory symptoms. There was no difference between treatment groups in non-ENT, nonrespiratory symptoms, such as those affecting the central nervous system, those affecting musculoskeletal tissues, or general fever or chills. This may suggest that niclosamide primarily exerts an effect via viral clearance from the respiratory tract, which deserves further investigation.

Our study enrolled outpatients who did not require hospitalization (1 participant, who was in the placebo group, progressed to severe disease). However, some participants were at increased risk for severe COVID-19, including those with BMI at 25 or more.^25^ In the niclosamide group, these individuals’ symptoms resolved in a mean time of 11.57 (95% CI, 6.92 to 16.23) days vs 16.46 (95% CI, 10.94 to 21.98) days in the placebo group, which was not a statistically significant difference.

A goal of this study was to understand the potential effects of niclosamide on fecal shedding of SARS-CoV-2. Oropharyngeal tissues and the intestine are established reservoirs for SARS-CoV-2. Examining viral loads from both sites may help to better estimate total viral burden, compared with standard oropharyngeal assessment alone. Across all participants in our study, the mean time to fecal SARS-CoV-2 clearance was 6.12 (95% CI, 3.51 to 8.73) days in the niclosamide group and 5.77 (95% CI, 3.3 to 8.23) days in the placebo group. Among patients with fecal shedding, mean time to fecal viral clearance in the niclosamide group was 13.21 (95% CI, 9.75 to 16.68) days vs 11.70 (95% CI, 8.47 to 14.93) days in the placebo group. These differences were not statistically significant. The principal mode by which people are infected with SARS-CoV-2 is through exposure to respiratory secretions carrying infectious virus, which can occur via inhalation or deposition of respiratory droplets on mucous membranes of the mouth, nose, or eye.^26^ The fecal-oral route has been postulated as well,^27^ but the retention of infectivity by fecally shed virus has been debated.^28,29,30^ Previous studies have found large quantities of SARS-CoV-2 by PCR in fecal samples from patients with COVID-19, potentially related to virus material produced in the airways being swallowed.^28^ Active replication of SARS-CoV-2 in the intestinal tract has also been reported.^31^ Our study used PCR-based analysis of cycle threshold values, often cited as the criterion standard for diagnosis of COVID-19 since the method’s authorization in February 2020^32^; however, viral plaque-forming assays would be a more direct measurement of infectious virions.^33^Future studies could be conducted to investigate if this fecal shedding is directly reflective of transmissibility or suggestive of oropharyngeal clearing of SARS-CoV-2 via the swallowing of virus-containing secretions and ultimate excretion in fecal samples, given that has been reported with similar respiratory viruses.^34^

### Limitations

Our study has several limitations. There was a precipitous and persistent decrease in the rate of COVID-19 diagnoses in spring and summer 2021 that decreased our enrollment pool. Additionally, as vaccination rates rose, our predetermined exclusion criteria of individuals vaccinated against SARS-CoV-2 limited those available for enrollment. An additional limitation of this study is the underenrollment of African American and Hispanic participants, given that these populations may be at increased risk of COVID-19 infection.^35,36^

Another limitation is that because this study was conducted remotely, drug blood levels were not available. We used the maximum conventional dose of niclosamide prescribed for dwarf worm infestation.^37^ Although the concentration required to achieve antiviral activity based on previous niclosamide studies^4,38^ is approximately 0.5 μM, which corresponds to 0.164 μg/mL, we do not know if participants achieved sufficient systemic drug concentration in our study. In a 2018 clinical trial to test efficacy of niclosamide as an antimetastatic therapy,^14,15^ clinicians found that upon oral intake, niclosamide Cmax plasma level peaked at a median (range) of 0.665 (0.429-0.848) μg/ml, suggesting that oral administration should be sufficient to inhibit SARS-CoV-2 production. Efficacy of niclosamide may be further enhanced by direct delivery through the respiratory tract as an inhaled medication. Given that niclosamide is historically well-tolerated, using the standard dosing regimen for parasitic treatment applications, future drug escalation studies should be considered to maximize antiviral effects against SARS-CoV-2.

Additionally, we cannot extrapolate our findings to patients with severe COVID-19. Although vaccines are still the first line of defense against COVID-19 given that they prevent progression to severe COVID-19 disease and death,^39^ niclosamide may warrant further investigation in a wider range of patients.

## Conclusions

There are increasing concerns about the efficacy of the current generation of COVID-19 vaccines against emergent SARS-CoV-2 variants based on the occurrence of breakthrough infections among fully vaccinated patients.^40^ Recent studies have demonstrated potent in vitro antiviral efficacy of niclosamide against the D614G, B.1.1.7, and B.1.351 variants, reinforcing its potential as a COVID-19 therapeutic.^5^ While our study did not find significant differences between niclosamide and placebo in viral clearing, it will be important to confirm these findings in a study enrolling a larger population of patients with COVID-19.

## Notes

Supplement 1.
eMethods.
**eTable.** Adverse Events Among All Participants by Treatment Group **eFigure 1.** Oropharyngeal and Fecal Shedding of SARS-Cov-2 by Treatment Group **eFigure 2.** Fecal Shedding of SARS-Cov-2 Among All Participants **eFigure 3.** Fecal Shedding of SARS-Cov-2 Among Participants With Fecal Shedding **eFigure 4.** Effect of Niclosamide on COVID-Related Symptoms Associated With the Central Nervous System **eFigure 5.** Effect of Niclosamide on COVID-Related Symptoms Associated With the Musculoskeletal System **eFigure 6.** Effect of Niclosamide on COVID-Related Fevers and Chills
eReferences.

Supplement 2.
Trial Protocol

Supplement 3.
Data Sharing Statement
