# Supplementary material for: Efficacy of Niclosamide vs Placebo in SARS-CoV-2 Respiratory Viral Clearance, Viral Shedding, and Duration of Symptoms Among Patients With Mild to Moderate COVID-19: A Phase 2 Randomized Clinical Trial
Source: JAMA Netw Open. 2022 Feb 9;5(2):e2144942. doi: 10.1001/jamanetworkopen.2021.44942 (PMC8829666; doi:10.1001/jamanetworkopen.2021.44942)
Supplement: Supplement 1. — eMethods. eTable. Adverse Events Among All Participants by Treatment Group eFigure 1. Oropharyngeal and Fecal Shedding of SARS-Cov-2 by Treatment Group eFigure 2. Fecal Shedding of SARS-Cov-2 Among All Participants eFigure 3. Fecal Shedding of SARS-Cov-2 Among Participants With Fecal Shedding eFigure 4. Effect of Niclosamide on COVID-Related Symptoms Associated With the Central Nervous System eFigure 5. Effect of Niclosamide on COVID-Related Symptoms Associated With the Musculoskeletal System eFigure 6. Effect of Niclosamide on COVID-Related Fevers and Chills eReferences. [file jamanetwopen-e2144942-s001.pdf]

Supplemental Online Content

Cairns DM, Dulko D, Griffiths JK, et al. Efficacy of niclosamide vs placebo in SARS-CoV-2 respiratory viral clearance, viral shedding, and duration of symptoms among patients with mild to moderate COVID-19: a phase 2 randomized clinical trial. *JAMA Netw Open*. 2022;5(1):e2144942. doi:10.1001/jamanetworkopen.2021.44942

eMethods.

eTable. Adverse Events Among All Participants by Treatment Group

eFigure 1. Oropharyngeal and Fecal Shedding of SARS-Cov-2 by Treatment Group

eFigure 2. Fecal Shedding of SARS-Cov-2 Among All Participants

eFigure 3. Fecal Shedding of SARS-Cov-2 Among Participants With Fecal Shedding

eFigure 4. Effect of Niclosamide on COVID-Related Symptoms Associated With the Central Nervous System

eFigure 5. Effect of Niclosamide on COVID-Related Symptoms Associated With the Musculoskeletal System

eFigure 6. Effect of Niclosamide on COVID-Related Fevers and Chills

eReferences.

This supplemental material has been provided by the authors to give readers additional information about their work.

© 2022 Cairns DM et al. *JAMA Network Open.*

eMethods.

Twice daily, a list of COVID-19 positive patients was generated by the Tufts Medical Center lab and securely provided to the study team. Upon receiving the positive test notification, COVID-19 positive participants were approached and screened by a study team member. Following initial screening for eligibility, a Health Insurance Portability and Accountability Act of (HIPAA) compliant telehealth platform was used to conduct a remote Informed Consent (IC) visit with a study team physician investigator.

For this study involving participants with COVID-19 positivity, the following steps were performed while obtaining the remote IC via telehealth:

- Purpose of the study and the potential risks/benefits of the use of the Investigational Agent (niclosamide) in the treatment of confirmed COVID-19 infection was discussed in detail
- Opportunity to review the IC form prior to or during the discussion was provided. Adequate time for discussion between the physician investigators was given to each potential participant.
- Availability and/or possibility of other potential treatment options were discussed.
- A second member of the study team was present during the entire IC discussion. The witness asked the potential participant if they understood the contents of the discussion and if they had any questions to address. The participant was informed that they could ask questions at any time during the trial.
- The participant and physician investigator and witness signed the IC form via a secure Docusign account.

During the screening process, information on all concomitant therapies, medications, and procedures were recorded in the source documents along with the diagnosis or reason for use. Those therapies used for the treatment of an adverse event (AE) were linked to an AE and documentation of the AE completed. Focused medical history based on known risks for severe COVID infection were obtained along with smoking history, demographics and record of .positive COVID-19 result by PCR were obtained. Clinical data, AEs, concomitant medications, and any other data collected from participants was entered into a REDCap database with password protection and internal quality checks, such as automatic range checks, to identify data that appear inconsistent, incomplete, or inaccurate.

Participants who provided informed consent and met all of the inclusion and none of the exclusion criteria were randomized on treatment Day 0 in a 1:1 ratio to either the Treatment Group (niclosamide) or the Control Group (placebo), in accordance with a computer-generated schedule. Study personnel were instructed not to randomize until participant had been confirmed to meet all inclusion/exclusion criteria on treatment Day 0. Following randomization, the investigational pharmacy packaged and dispensed either niclosamide or placebo, dispensing the entire supply at one time. Participants in the Treatment Group received niclosamide 2 grams orally once daily for 7 days in addition to current standard of care treatment. Those in the Control Group received placebo by mouth in the same numbers of pills for 7 days in addition to current standard of care treatment. In addition to niclosamide or placebo, all enrolled participants were provided an oral thermometer and fingertip probe pulsoximeter with the specific instructions to monitor and record both temperature at oxygen saturation at the time of daily oral administration of drug. All study materials were delivered to participants via courier service.

1.1 Sample Size Calculation

For the primary efficacy endpoint of respiratory virologic clearance at Day 3 measured by oropharyngeal viral shedding, 40 participants in each group achieve 89.1% power to detect a difference between the group proportions of 35%. We assumed that 50% and 15% of participants in the niclosamide and placebo groups would have a negative test on Day 3. The calculation was under a two-sided Fisher's Exact Test and a significance level of 0.05.

© 2022 Cairns DM et al. *JAMA Network Open.*

1.2 Inclusion and Exclusion Criteria

Inclusion Criteria

- SARS-CoV-2 infection confirmed by PCR ≤ 3 days before randomization
- Provision of informed consent
- Stated willingness to comply with all study procedures and availability for the duration of the study
- Male or female, over 18 years of age
- No need for supplemental oxygen
- Temperature ≥ 36.6 °C axilla, ≥ 37.2 °C oral, or ≥ 37.8 °C rectal
- No requirement for hospitalization at the time of enrollment
- Ability to take oral medication and be willing to adhere to the niclosamide/placebo regimen

Exclusion Criteria

- Known allergic reactions to components of the niclosamide
- History of receipt of COVID-19 vaccine
- Participation in another trial or use of any experimental treatment for COVID-19, including chloroquine, hydroxychloroquine, remdesivir, and lopinapir/ritonavir
- Current hospitalization or requiring hospital admission at screening

On treatment Day 1, a member of the study team reviewed the dosing and schedule of niclosamide/placebo instructing the participant to record all doses in Study Drug Administration Diary. Baseline symptoms, oxygen saturation, temperature, and observed oropharyngeal sample collection was obtained.

Fecal samples and oropharyngeal swab samples were collected for viral shedding as measured by PCR on days 1, 3, 7, 10, 14 and 21 (day 21 - fecal sample only). The collection of specimens was directly observed by a study team member via the telehealth platform educating the participant to open the viral transport kit and swab the back of the throat and tonsil area (avoiding mouth, teeth, and gums) and place to swab back into the viral media vial. For fecal specimens, the participant was instructed to swab feces from a plastic container (or wrap) placed on the toilet seat. Printed instructions were provided to each participant with detail on how to collect both the oropharyngeal and fecal specimens. These instructions were reviewed with the participant by a member of the study team at the start of the Day 1 telehealth visit.

Once obtained, samples were transported by FedEx service to .the Clinical Laboratory Improvement Amendments of 1988 (CLIA)-certified laboratory to avoid unnecessary hospital visits and to encourage compliance, given the self-quarantine status of enrolled patients.

Remote clinical follow-up via telehealth was performed at the following time points: Days 2, 3, 7, 10, 14, 21 and 30 including the following:

- Obtaining AE data
- Recording patient-reported COVID signs and symptoms
- Documentation of patient's status as an outpatient, subsequently hospitalized, or died
- Documentation of patient reported and vital signs (temperature and oxygen saturation)
- Evaluate for increased severity of COVID-19-related disease
- Collection of temperature and oximetry data

1.3 Timing of outcome assessments, including visit windows

Primary efficacy endpoint: respiratory viral clearance at Day 3.

Secondary efficacy endpoints

- Fecal viral clearance at Day 14.

© 2022 Cairns DM et al. *JAMA Network Open.*

- Reduction in viral shedding as measured by oropharyngeal swab on days, 3, 7, 10, 14.
- Reduction in fecal viral shedding as measured by fecal PCR on days, 3, 7, 10, 14 and 21.
- Progression to severe COVID, as a composite endpoint defined as O2 saturation <92% on room air in two consecutive measurements at least 2 hours apart OR requirement of hospitalization OR need for artificial ventilation OR death.
- Resolution of symptoms

1.4 Adherence and protocol deviations

In this study, four pills are taken daily for 7 days. Compliance is assessed by the percentage of subjects who have taken the scheduled number of pills:

% compliance = 100x (number of pills taken / 28 pills prescribed). Compliance is summarized by randomization group: mean % compliance as well as number and percentage of participants with more than 80% compliance (24 pills out of 28). Similarly, compliance with OP and fecal sampling was calculated.

1.5 Analysis populations

Participants who withdrew consent after randomization but before taking any sample or pill were excluded. The intention-to-treat (ITT) population includes all randomized patients according to the treatment they were randomized to receive (niclosamide or placebo). The per-protocol population is a subset of the participants in the full analysis (ITT) set who took at least 80% of study intervention. The safety population includes participants who took at least one pill.

1.6 Statistical Analyses

All participants (n=67) who attended the first telehealth visit were included in analyses. The cumulative probability of viral clearance in each group was estimated using the Kaplan-Meier estimator. Viral clearance was met the first day a participant's sample result was negative, provided that none of the subsequent sample results were positive. The starting time for survival analyses was the day of the first telehealth visit. If clearance was not met, the participant was censored at the time of the last available sample. For the primary analysis, the cumulative probabilities of clearance, based on oropharyngeal samples, was compared between treatment groups at Day 3 using a chi-square test based on the log of the -log transformation for the survival function[1]. Kaplan-Meier plots of cumulative probability of clearance were created and the log-rank test was used to compare the curves. Mean time to clearance up to day 14 for analyses involving respiratory samples and day 21 for analyses involving fecal samples were calculated using the area under the clearance-free survival curve.

These analyses were also utilized to compare time to symptom resolution between treatment groups. Subgroup analyses of single shedders (without fecal shedding) vs. double shedders (with fecal shedding), with double-shedders defined as participants with a positive test for at least one stool sample, used the same statistical techniques. All statistical tests were 2-sided, performed using a 5% significance level, and 95% two-sided confidence intervals were calculated.
All analyses were performed using SAS Enterprise Guide and R.

© 2022 Cairns DM et al. *JAMA Network Open.*

eTable. Adverse Events Among All Participants by Treatment Group

Adverse Event	Placebo (N=34)	Niclosamide (N=33)	Overall (N=67)
**Abdominal Pain**	3 (8.8)	1 (3.0)	4 (6.0)
**Congestion or runny nose**	4 (11.8)	8 (24.2)	12 (17.9)
**Cough**	8 (23.5)	7 (21.2)	15 (22.4)
**Diarrhea**	4 (11.8)	5 (15.2)	9 (13.4)
**Dizziness**	2 (5.9)	1 (3.0)	3 (4.5)
**Dyspnea**	0 (0.0)	2 (6.1)	2 (3.0)
**Fatigue**	4 (11.8)	6 (18.2)	10 (14.9)
**Fever or chills**	3 (8.8)	1 (3.0)	4 (6.0)
**Headaches**	11 (32.4)	7 (21.2)	18 (26.9)
**Hypoxia**	1 (2.9)	0 (0.0)	1 (1.5)
**Loss of appetite**	1 (2.9)	0 (0.0)	1 (1.5)
**Muscle or body aches**	3 (8.8)	1 (3.0)	4 (6.0)
**Nausea**	7 (20.6)	2 (6.1)	9 (13.4)
**New loss of taste or smell**	5 (14.7)	2 (6.1)	7 (10.4)
**Pruritus**	0 (0.0)	1 (3.0)	1 (1.5)
**Shortness of breath, difficulty breathing**	2 (5.9)	5 (15.2)	7 (10.4)
**Skin rash**	3 (8.8)	3 (9.1)	6 (9.0)
**Sore throat**	0 (0.0)	2 (6.1)	2 (3.0)
**Vomiting**	2 (5.9)	0 (0.0)	2 (3.0)

© 2022 Cairns DM et al. *JAMA Network Open.*

eFigure 1. Oropharyngeal and Fecal Shedding of SARS-Cov-2 by Treatment Group

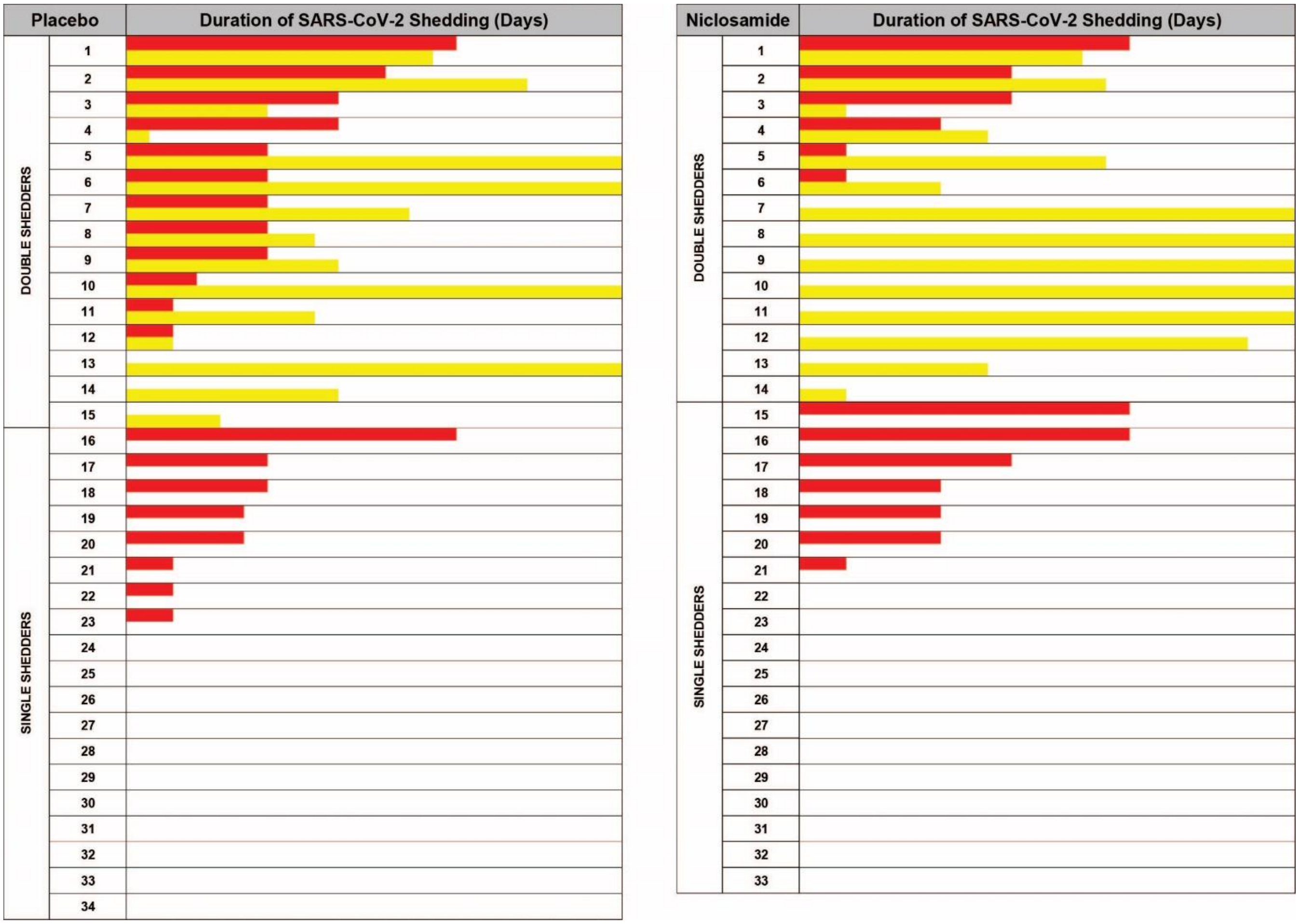

Oropharyngeal and fecal shedding of SARS-CoV-2 in individual participants receiving niclosamide or placebo. “Single shedders" refers to those participants without fecal shedding, and "double shedders" refers to those participants with fecal shedding. Visual representation of timeline of SARS-CoV-2 shedding in oropharyngeal (red bars) and fecal (yellow bars) samples across treatment groups.

© 2022 Cairns DM et al. *JAMA Network Open.*

eFigure 2. Fecal Shedding of SARS-Cov-2 Among All Participants

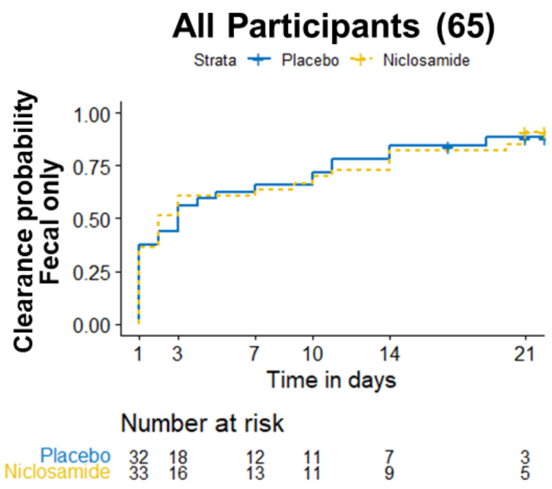

The mean time to fecal clearance of SARS-CoV-2 in all participants up to Day 21 was 6.12 days (95%CI 3.51 to 8.73) in the niclosamide group and 5.77 days (95%CI 3.3 to 8.23) in the placebo group, mean difference 0.36 days (95%CI -3.23 to 3.95).

© 2022 Cairns DM et al. *JAMA Network Open.*

eFigure 3. Fecal Shedding of SARS-Cov-2 Among Participants With Fecal Shedding

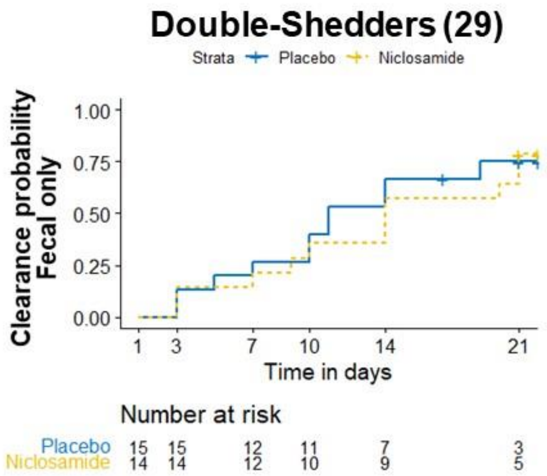

The mean time to fecal clearance of SARS-CoV-2 in .the double shedder population (participants with fecal shedding) up to Day 21 was 13.21 days (95%CI 9.75 to 16.68) in the niclosamide group and 11.7 days (95%CI 8.47 to 14.93) in the placebo group, mean difference 1.51 days (95%CI -3.22 to 6.25).

© 2022 Cairns DM et al. *JAMA Network Open.*

eFigure 4. Effect of Niclosamide on COVID-Related Symptoms Associated With the Central Nervous System

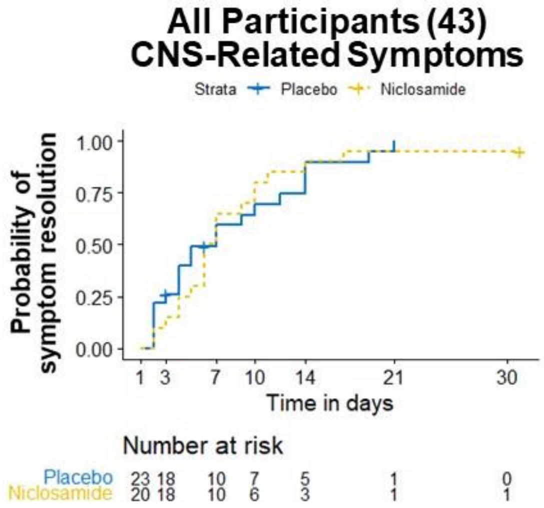

Symptoms associated with the central nervous system (CNS) include dizziness, fatigue and headache. The mean time to CNS-related symptom resolution up to Day 30 was 7.3 days (95%CI 4.57 to 10.03) in the niclosamide group and 7.04 days (95%CI 4.59 to 9.48) in the placebo group, mean difference 0.26 days (95%CI -3.4 to 3.93).

© 2022 Cairns DM et al. *JAMA Network Open.*

eFigure 5. Effect of Niclosamide on COVID-Related Symptoms Associated With the Musculoskeletal System

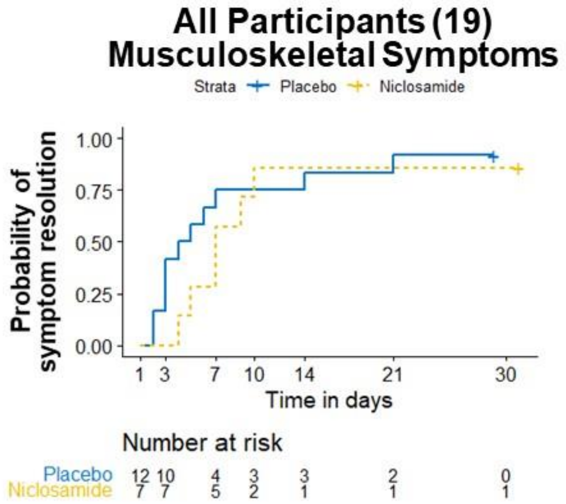

Symptoms associated with the musculoskeletal system include muscle aches. The mean time to musculoskeletal-related symptom resolution up to Day 28 was 9.14 days (95%CI 3.26 to 15.02) in the niclosamide group and 7.25 days (95%CI 2.57 to 11.93) in the placebo group, mean difference 1.89 days (95%CI -5.62 to 9.41).

© 2022 Cairns DM et al. *JAMA Network Open.*

eFigure 6. Effect of Niclosamide on COVID-Related Fevers and Chills

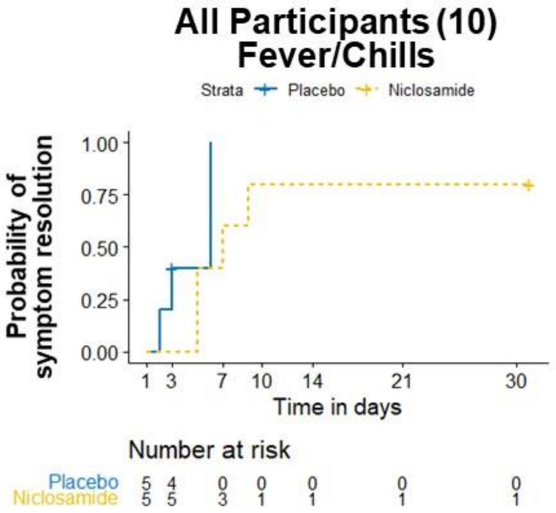

The mean time to resolution of fever/chills up to Day 30 was 10.2 days (95%CI 1.86 to 18.54) in the niclosamide group and 3.6 days (95%CI 2.07 to 5.13) in the placebo group, mean difference 6.6 days (95%CI -1.88 to 15.08).

© 2022 Cairns DM et al. *JAMA Network Open.*

eReferences

1. Klein, J.P., et al., *Analyzing survival curves at a fixed point in time.* Statistics in Medicine, 2007. **26**(24): p. 4505-4519.

© 2022 Cairns DM et al. *JAMA Network Open.*
