# Supplementary material for: Efficacy of Niclosamide vs Placebo in SARS-CoV-2 Respiratory Viral Clearance, Viral Shedding, and Duration of Symptoms Among Patients With Mild to Moderate COVID-19: A Phase 2 Randomized Clinical Trial
Source: JAMA Netw Open. 2022 Feb 9;5(2):e2144942. doi: 10.1001/jamanetworkopen.2021.44942 (PMC8829666; doi:10.1001/jamanetworkopen.2021.44942)
Supplement: Supplement 2. — Trial Protocol [file jamanetwopen-e2144942-s002.pdf]

Niclosamide for Patients with Mild to Moderate Disease

from Novel Coronavirus (COVID-19)

Principal Investigator: Harry Selker, MD

01202021

Summary of Changes from Previous Version:

Affected Section(s)	Summary of Revisions Made	Rationale
Protocol Title	Addition of asymptomatic patients	1. FDA recommendation
Study Synopsis	Addition of Day 21 fecal specimen, addition of asymptomatic participants, addition of Wellforce and Clinical Research Network (CRN) sites for recruitment.<br>Study duration 2 months, study completion 4 months	1. FDA recommendation<br>2. Recruitment optimization<br>3. Decreased numbers of daily COVD positive patients
Section 3.0 Objectives and endpoints	The primary objective of this study is to determine if a course of treatment with niclosamide improves respiratory viral clearance compared to treatment with placebo.	1. Viral shedding endpoint
Section 4.0 Study design	Reduction in fecal viral shedding as measured by fecal PCR on days, 3, 7, 10, 14. Day 21 fecal specimen added.	1. FDA recommendation
Section 4.3 Justification of Dose	Additional literature and background added	1. FDA recommendation
Section 5.1 Inclusion Criteria	Physical assessment data removed. No need for oxygen supplementation added.	1. Virtual recruitment
5.2 Exclusion Criteria	Systemic treatments removed as exclusion.	1. FDA recommendation
5.3 Lifestyle considerations	Advisement to avoid alcohol added	1. FDA recommendation
5.5 Strategies for recruitment and retention	1. Updates to include Wellforce and CRN sites<br>2. Twenty-dollar ($20) ClinCard payment at each specimen collection timepoint<br>3. Update to recruitment with community outreach, study information distribution and nurses script	1. Recruitment enhancement<br>2. Time and effort of participants for specimen collection

6.1.1. Study Intervention	Day 21 fecal specimen added<br>Additional data on oropharyngeal specimen collection added	1. FDA recommendation
6.2.2 Formulation, Appearance, Packaging and Labeling	Study drug specific information added as provided by Bayer Pharmaceuticals	1. Updated as per IND
6.2.3 and 6.2.4 Product storage and preparation	Changed to blister packaging	1. Updated as per IND
7.2 Participant discontinuation/ withdrawal from study	Revised	1. FDA recommendation
8.1 Efficacy assessments	Baseline and screening (Day 0)<br>Record COVID symptoms, if symptomatic<br><br>Record number of days since onset of symptoms, if symptomatic<br><br>Follow-up Evaluation<br><br>• Collect temperature and oximetry data daily, with the caveat that if O2 goes below 92%, an addition oximeter reading should be taken 2 hours later. ADDED: If the follow-up O2 saturation remains below 92%, the participant will be referred to their Primary Care Physician (PCP) or to the local Emergency Department<br><br>•Collect fecal samples for viral shedding Day 21 added<br><br>**Early Termination/Hospitalization**<br><br>A 30-day follow-up call and AE	1. FDA recommendation

	assessment for those patients who are hospitalized will be performed.	
8.3.7 Reporting of pregnancy	Additional information added re: pregnancy and breast feeding	1. FDA recommendation
10.1.6 Safety Oversight	Independent Safety Monitor and stopping rules added	1. FDA recommendation
4.1 Overall Study Design	Addition of affiliate site recruitment detail	1. Reliance affiliate
1.1 Study Synopsis	Primary endpoint change	1. Primary Efficacy Endpoint: Respiratory viral clearance at Day 3.
1.2 Schedule of activities	Addition of +/1 1-day windows	1. Increase options for participant study visits
4.1 Overall study design	Increase sample size to n=200	1. The current positivity rate on Day 1 of ~40% results in ITT sample size of 200.
5.1 Exclusion Criteria	Add: History of receipt of COVID-19 vaccine	1. Potential effect of vaccine on viral shedding
5.2 Strategies for Recruitment	Added: Newton Wellesley Hospital, Maine Medical Center and New England Quality Care Alliance (NEQCA)	1. Agreed to distribute study brochures and posters

6.1 Study Intervention	Removed: Transport by courier service to the Tufts Medical Center<br><br>Telemedicine Platform<br><br>Removed: Amwell<br><br>Added: A HIPPA compliant telehealth platform; e.g., Doximity, will be used to conduct remote study visits.<br><br>Added: COVID positive list generated twice daily	1. No contact FedEx pickup/ delivery to CLIA certified lab<br>2. Doximity platform used
9.1 Statistical Hypothesis	Sample size calculation revision	1. Sample size increase
9.2 Sample Size Determination	Revised	1. Increase d sample size
9.3 Populations for analysis	Modified Intention-to-Treat Analysis Dataset modified	1. Sample size increase
9.4 Statistical Analysis	Revised analysis of the primary endpoint	1. Sample size increase

Table of Contents

STATEMENT OF COMPLIANCE 1
1 PROTOCOL SUMMARY 1
1.1 Synopsis 1
SchemA 3
1.2 Schedule of Activities (SoA) 3
2 INTRODUCTION 5
2.1 Study Rationale 5
2.2 Background 6
2.3 Risk/Benefit Assessment 7
2.3.1 Known Potential Risks 7
2.3.2 Known Potential Benefits 7
2.3.3 Assessment of Potential Risks and Benefits 7
3 OBJECTIVES AND ENDPOINTS 8
4 STUDY DESIGN 8
4.1 Overall Design 8
4.2 Scientific Rationale for Study Design 10
4.3 Justification for Dose 10
4.4 End of Study Definition 11
5 STUDY POPULATIOn 11
5.1 Inclusion Criteria 12
5.2 Exclusion Criteria 12
5.3 Lifestyle Considerations 12
5.4 Screen Failures 12
5.5 Strategies for Recruitment and Retention 12
6 STUDY INTERVENTION 13
6.1 Study Intervention(s) Administration 13
6.1.1 Study Intervention Description 13
6.1.2 Dosing and Administration 15
6.2 Preparation/Handling/Storage/Accountability 15
6.2.1 Acquisition and accountability 15
6.2.2 Formulation, Appearance, Packaging, and Labeling 16
6.2.3 Product Storage and Stability 16
6.2.4 Preparation 16
6.3 Measures to Minimize Bias: Randomization and Blinding 16
6.4 Study Intervention Compliance 16
6.5 Concomitant Therapy 17
6.5.1 Rescue Medicine 17
7 STUDY INTERVENTION DISCONTINUATION AND PARTICIPANT DISCONTINUATION/WITHDRAWAl. 17
7.1 Discontinuation of Study Intervention 17
7.2 Participant Discontinuation/Withdrawal from the Study 18
7.3 Lost to Follow-Up 18
8 STUDY ASSESSMENTS AND PROCEDURES 19
8.1 Efficacy Assessments 19
8.2 Safety and Other Assessments 21
8.3 Adverse Events and Serious Adverse Events 21

8.3.1 Classification of an Adverse Event. 22
8.3.2 Time Period and Frequency for Event Assessment and Follow-Up 23
8.3.3 Adverse Event Reporting 23
8.3.4 Serious Adverse Event Reporting 23
8.3.5 Reporting Events to Participants 24
8.3.6 Events of Special Interest 24
8.3.7 Reporting of Pregnancy 24
8.4 Unanticipated Problems 24
8.4.1 Definition of Unanticipated Problems (UP): 24
8.4.2 Unanticipated Problem Reporting 24
8.4.3 Reporting Unanticipated Problems to Participants 25
9 STATISTICAL CONSIDERATIONS 25
9.1 Statistical Hypotheses 25
9.2 Sample Size Determination 25
9.3 Populations for Analyses 25
9.4 Statistical Analyses 26
9.4.1 General Approach 26
9.4.2 Analysis of the Secondary Endpoint(s) 27
9.4.3 Safety Analyses 27
9.4.4 Baseline Descriptive Statistics 27
9.4.5 Planned Interim Analyses 28
9.4.6 Sub-Group Analyses 28
9.4.7 Tabulation of Individual participant Data 28
9.4.8 Exploratory Analyses 28
10 SUPPORTING DOCUMENTATION AND OPERATIONAL CONSIDERATIONS 28
10.1 Regulatory, Ethical, and Study Oversight Considerations. 28
10.1.1 Informed Consent Process 28
10.1.2 Study Discontinuation and Closure 30
10.1.3 Confidentiality and Privacy 30
10.1.4 Future Use of Data 31
10.1.5 Key Roles and Study Governance 31
10.1.6 Safety Oversight 32
10.1.7 Clinical Monitoring 33
10.1.8 Quality Assurance and Quality Control 34
10.1.9 Data Handling and Record Keeping 34
10.1.10 Protocol Deviations 35
10.1.11 Publication and Data Sharing Policy 35
10.1.12 Conflict of Interest Policy 35
10.2 Additional Considerations 36
10.3 Abbreviations 37
10.4 Protocol Amendment History 39
11 REFERENCES 40

STATEMENT OF COMPLIANCE

The trial will be carried out in accordance with International Conference on Harmonisation Good Clinical Practice (ICH GCP) and the following:

- United States (US) Code of Federal Regulations (CFR) applicable to clinical studies (45 CFR Part 46, 21 CFR Part 50, 21 CFR Part 56, 21 CFR Part 312, and/or 21 CFR Part 812)

National Institutes of Health (NIH)-funded investigators and clinical trial site staff who are responsible for the conduct, management, or oversight of NIH-funded clinical trials have completed Human Subjects Protection and ICH GCP Training.

The protocol, informed consent form(s), recruitment materials, and all participant materials will be submitted to the Institutional Review Board (IRB) for review and approval. Approval of both the protocol and the consent form must be obtained before any participant is enrolled. Any amendment to the protocol will require review and approval by the IRB before the changes are implemented to the study. In addition, all changes to the consent form will be IRB-approved; a determination will be made regarding whether a new consent needs to be obtained from participants who provided consent, using a previously approved consent form.

1 PROTOCOL SUMMARY

1.1 SYNOPSIS

Title: **Niclosamide for Patients with Mild to Moderate Disease from Novel Coronavirus (COVID-19)**

Study Description: This study will evaluate the antihelmintic drug, niclosamide, as a potential treatment for mild to moderate coronavirus disease 2019 (COVID-19). Niclosamide, which has potent antiviral activity against single-stranded RNA viruses including coronaviruses, was proposed as an antiviral during the SARS outbreak in 2002 and has activity including SARS-CoV-2 where it was found to inhibit SARS coronavirus, SARS-CoV, in *in vitro* studies and similarly structured RNA viruses (both *in vitro* and *in vivo)*. We hypothesize that the antiviral activity of Niclosamide may be extended to COVID-19.

Objectives:

- Primary Objective: To evaluate the efficacy of niclosamide in shortening contagious period as determined by time to viral clearance.
- Secondary Objectives: To evaluate the efficacy of niclosamide in mitigating clinical outcomes and shortening duration of symptoms resulting from COVID-

19 infection.

Endpoints:

- Primary Efficacy Endpoint: Respiratory viral clearance at Day 3.
- Secondary Efficacy Endpoints:
  - Fecal viral clearance at Day 14.
  - Reduction in viral shedding as measured by oropharyngeal swab on days 1, 3, 7, 10, 14
  - Reduction in fecal viral shedding as measured by fecal PCR on days 1, 3, 7, 10, 14, 21
  - Progression to severe COVID, as a composite endpoint defined as O2 saturation <92% on room air in two consecutive measurements at least 2 hours apart OR requirement of hospitalization OR need for artificial ventilation OR death.
  - Time to resolution of fever

Safety endpoint: incidence of Adverse Events (AEs)

Study Population: Patients 18 years of age or older who are COVID-19 (SARS-CoV-2) positive by PCR who are asymptomatic or have mild to moderate symptoms of COVID infection.

Phase: Phase II

Description of Sites/Facilities Study participants will be recruited for participation from Tufts Medical Center, Wellforce and Clinical Research Network (CRN) sites.

Enrolling Participants: Participants will be identified as those reporting to Tufts Medical Center, Wellforce and CRN sites for outpatient COVID-19 testing. Patients with SARS-CoV-2 positive test results will be provided the option to participate in our study. The Study Team will enroll and randomize patients into the study after the patient has a confirmed positive test result and meets all the inclusion and none of the exclusion criteria.

Additional sites and/or social media may be used to enhance recruitment. The study will be listed at *www.clinicaltrials.gov.*

Description of Study Intervention: Participants in the treatment arm will receive niclosamide 2 grams orally once daily for 7 days in addition to current standard of care treatment. Those in the control group will receive placebo by mouth in the same numbers of pills on day 1 and daily for 6 more days (total 7 days of treatment) in addition to current standard of care treatment. Fecal samples and oropharyngeal swab samples will be collected for viral shedding as measured by PCR on days 3, 7, 10, 14 and 21 (fecal sample only). A baseline fecal and oropharyngeal sample will be obtained on Day 1 prior to starting dosing of niclosamide/ placebo.

Study Duration: The study is estimated to complete enrollment within 2-months of initiation of enrollment; however, enrollment will remain open until the study goal is met. The duration of the entire project is anticipated to be a maximum of 4 months.

Participant Duration: Individual participant will complete all participant visits within 30 days. Adverse events will be monitored and collected by the Study Team from the point of signed consent until 7 (for non-serious adverse events) or 30 days (for serious adverse events) after the last day of study participation.

SCHEMA

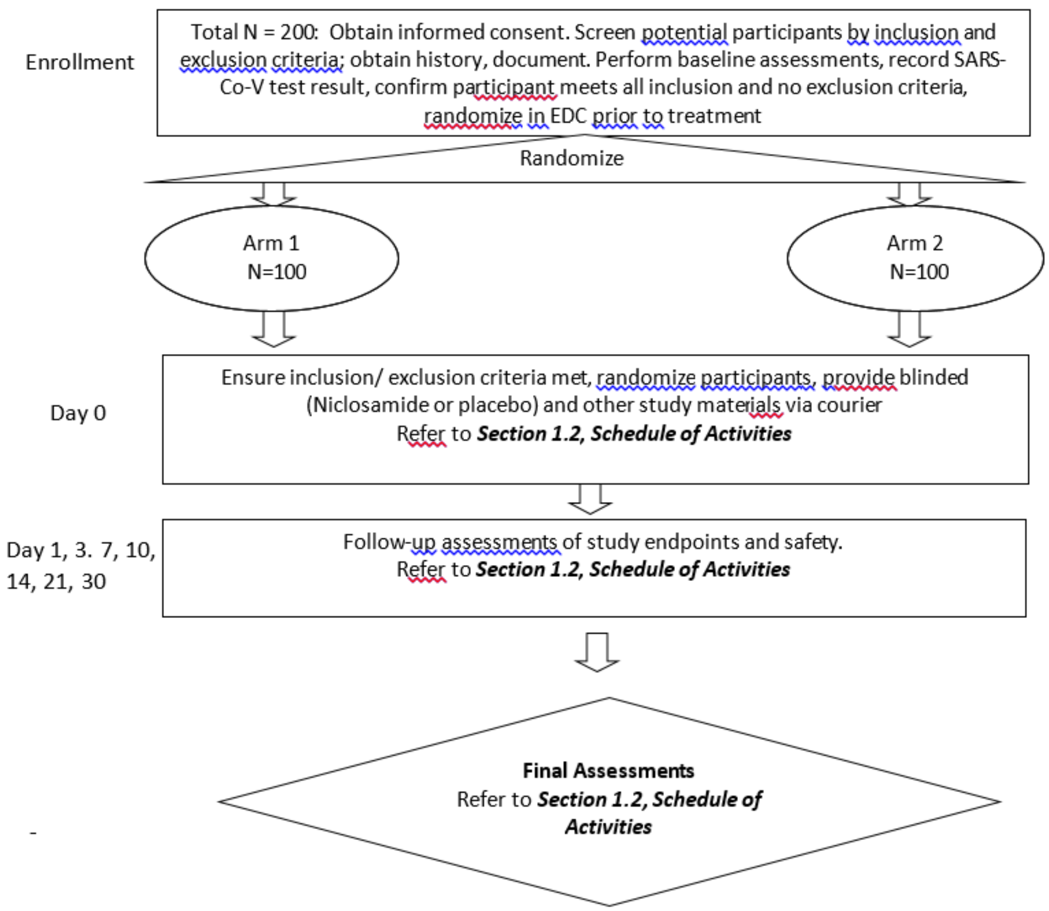

1.2 SCHEDULE OF ACTIVITIES (SOA)

	Screen & Baseline Day 0	Treat Day 1 +/- 1 day	Treat Day 2 +/- 1 day	Treat Day 3 +/- 1 day	Treat Days 4-6 +/- 1 day	Post-Treatment Day 7 +/- 1 day	Post-Treatment Day 10 +/- 1 day	Post-Treatment Day 14 * +/- 1 day	30-day Safety Call +/ - 1 Day
**Confirmed COVID-19 + test**	X								
**Medical/ Social History (Demographics)**	X								
**Inclusion/ Exclusion Criteria**	X								
**Informed Consent**	X								
**Assign Subject ID**	X								
**Randomize**	X								
	**Initiation of Study**								
**Dose treatment group with 2g niclosamide or placebo**		X	X	X	X				
**Provide sample collection packet, thermometer, and finger-tip pulse oximetry**		X							
	**Participant reporting and sample collection**								
**AE reporting**		X	X	X	X	X	X	X	X
**O2 & Temp reporting**		X	X	X	X	X	X	X	X
**Oropharyngeal & fecal sample**		X (prior to		X		X	X	X	

**collection**		**dosing)**							

* An additional AE assessment and fecal specimen will be collected at day 21

2 INTRODUCTION

2.1 STUDY RATIONALE

The ongoing COVID-19 pandemic is an urgent public health crisis with few if any rapid and practical solutions. The outbreak of COVID-19 has been declared to be a public health emergency of international concern by the World Health Organization (WHO), and the development of effective therapies for fast-spreading fatal COVID-19 is in an urgent need. Given the seriousness and time-sensitive nature of this highly contagious virus, the medical and scientific communities must work quickly and efficiently to find a feasible way to address this global emergency.

Niclosamide is an anthelminthic drug that has been widely used in humans to treat tapeworm infections for several decades and is currently listed on the WHO List of Essential Medicines, the safest and most effective medicines needed in a health system. There are no current proven treatments for COVID-19, and significant efforts are going toward developing novel therapeutics that have not been assessed for safety in humans. There are a number of existing drugs prescribed for other indications that have demonstrated potent antiviral activity [1-3]. In a recent study, Niclosamide exhibited antiviral activity against SARS-CoV-2, the strain responsible for the current COVID-19 pandemic [3]. That niclosamide has already demonstrated efficacy in specifically inhibiting SARS-CoV-2 replication in vitro is incredibly promising.

A recent study evaluated clinical samples from 73 hospitalized patients with SARS-CoV-2 infection. In 39 of those patients, their fecal samples tested positive for SARS-CoV-2 RNA, with 17 of those patients remaining SARS-CoV-2-positive in feces after becoming negative in respiratory samples [29]. Taken together, this suggests that both the commonly accepted route of infection through the respiratory system as well as the GI tract are implicated in the pathogenesis of COVID-19-related disease manifestations.

While severe acute respiratory syndrome (SARS) coronavirus 2, SARS-CoV-2-infected patients most commonly present with fever, tiredness and dry cough; a subset of these patients present with gastrointestinal (GI) issues [2]. Importantly, a recent study found that fecal viral shedding continues nearly 5 weeks after the last detection of SARS-CoV-2 RNA in respiratory samples, suggesting that the GI tract serves as a viral reservoir and allows for prolonged COVID-19 infection and transmission [4]. Given that SARS-CoV-2 is so highly contagious and can be easily spread by both respiratory droplets and fecal-oral route [5], limiting its transmission is paramount to public health. These data suggest that there is a critical need to develop practical COVID-19 intervention strategies to treat SARS-CoV-2 infection and to prevent person-to-person transmission.

Repurposing reliable and effective drugs for COVID-19 therapy is not only a safer strategy but will also allow for more rapid introduction into clinical practice. For this reason, we propose to use the widely used antihelmintic drug, Niclosamide for treatment of COVID-19.

2.2 BACKGROUND

SARS-CoV-2 has been shown to invade human tissues via the angiotensin converting enzyme II receptor (ACE2), which is highly expressed on cell types found in various tissues [30]. In a study to identify potential routes of infection for the SARS-CoV virus corresponding to the outbreak in China in 2002, a remarkable finding was the high surface expression of ACE2 protein on human lung alveolar epithelial cells and enterocytes, the simple columnar epithelial cells lining the inner surface of the small and large intestines[30].

This is not the first time that this small molecule (niclosamide) has been proposed as a therapeutic for this specific application. Shortly after the SARS outbreak in China in 2002-2003, niclosamide was tested for its potential use as an antiviral medication. Perhaps somewhat surprisingly, niclosamide was found to inhibit SARS coronavirus, SARS-CoV, in in vitro studies [14]. Later studies went on to evaluate its potential in combating coronavirus in vivo [31]. Fortunately, the SARS outbreak subsided rather quickly, and as a result, no future interventions were necessary.

Multiple studies have studied the antiviral capacity of niclosamide in treating other similarly structured pathogenic viruses. During the outbreak of the Zika virus (ZIKV), another positive-sense RNA-based virus similar to the coronavirus family, Niclosamide was again identified as a potential antiviral therapeutic, with ZIKV-inhibiting effects both in vitro [13] as well as in a humanized in vivo model of ZIKV-induced microcephaly [5]. Upon reviewing the literature, we found that niclosamide was also able to inhibit production of a variety of viral strains [4, 7, 8, 10-12, 15, 32], including adenovirus [10], dengue [4] and chikungunya virus [11]. Its mechanism of action in this capacity is to increase the pH within acidic endosomes of host cells, thereby inhibiting virus entry and release.

In addition to its antihelmintic and antiviral properties, niclosamide has also demonstrated anti-bacterial [8, 33, 34], anti-inflammatory [16, 17] and anti-cancer activity [18, 19, 23, 33, 35-40]. Moreover, niclosamide has also shown promise for treating respiratory illness [14, 18, 20-22] even functioning as a bronchodilator in an in vivo mouse model of asthma [21, 22].

Niclosamide has demonstrated efficacy as a cancer therapeutic in both animal models [19, 36] as well as human clinical trials [23, 35] which suggests that in addition to potentially being efficacious in this anti-COVID-19 capacity, Niclosamide is also likely to induce few complications as it is tolerated well even in immunocompromised cancer patients.

In the current pandemic crisis, medical professionals are understandably focused more on stabilizing the very sick. However, if niclosamide could work in any way to halt or prevent infection in the less sick (or even in the uninfected), it would be monumental in terms of returning to normal life. Because the drug is inexpensive and has few if any side effects, taking Niclosamide prophylactically might help to prevent COVID-19 spreading. Even if this treatment does not completely eradicate infection, niclosamide

treatment may help to decrease viral load, thereby allowing the host immune system to better combat the disease.

2.3 RISK/BENEFIT ASSESSMENT

2.3.1 KNOWN POTENTIAL RISKS

Niclosamide has been used since mid of the 1960s as an antihelmintic drug which inhibits glucose uptake, oxidative phosphorylation and anaerobic metabolism. Niclosamide has few side effects and is known to be well tolerated even when applied over a long period [19]. Reported adverse effects of Niclosamide are mild and infrequent. This may include GI disturbances, lightheadedness, malaise, and pruritus. Alcohol may enhance the absorption of niclosamide, increasing the risk of side effects and therefore should be avoided when taking this drug.

Pregnancy and breastfeeding are not exclusion criteria. The FDA categorizes medications based on safety for use during pregnancy. Five categories - A, B, C, D, and X, are used to classify the possible risks to an unborn baby when a medication is taken during pregnancy.

Niclosamide falls into category B:
• There are no well-done studies that have been done in humans with Niclosamide. But in animal studies, pregnant animals were given this medication, and the babies did not show any medical issues related to this medication.
• Studies in women suggest that this medication poses minimal risk to the infant when used during breastfeeding.

2.3.2 KNOWN POTENTIAL BENEFITS

Niclosamide is an oral medication that has been used to treat tapeworm infestations since 1960[6]. It is on the World Health Organization (WHO) List of Essential Medicines, the safest and most effective medicines needed in a health system [6]. In addition to its originally prescribed use, Niclosamide has been repurposed for a variety of clinical applications. Niclosamide has demonstrated antiviral activity both in vitro and in vivo on a variety of single stranded RNA-based viral strains [4, 5, 7-13] and even SARS-CoV, a previous strain of coronavirus associated with the SARS outbreak in China in 2002[14]. Even more recently, Niclosamide was shown to inhibit SARS-CoV-2, the strain responsible for the current COVID-19 pandemic [3]. Its mechanism of action in this capacity is to increase the pH within acidic endosomes of host cells, thereby inhibiting virus entry and release [4, 11, 15]. In addition to its antihelmintic and antiviral properties, Niclosamide has also demonstrated anti-inflammatory activity [16, 17], and has shown promise for treating respiratory illness [18-20] even functioning as a bronchodilator in an in vivo mouse model of asthma [21, 22]. Furthermore, that Niclosamide has been utilized in a variety of human clinical trials to enhance chemotherapeutics in cancer treatment [19, 23-25] suggests that is likely to induce few complications as it is tolerated well even in immunocompromised cancer patients.

2.3.3 ASSESSMENT OF POTENTIAL RISKS AND BENEFITS

The primary goal is to maximize patient safety. To fulfill this goal, Tufts and the study team will take every measure to reduce patient risk of COVID-19-related complications, which includes the following:

- Reduction of in-patient and outpatient face- to face visits to limit amount of exposure to patient and related populations
- Use of a courier service to provide study materials and no pickup and delivery FedEx to obtain patient samples required for this study
- Extensive patient screening prior to enrollment to ensure that all enrolled patients meet inclusion/exclusion criteria; more specifically to avoid enrollment of COVID-19 patients already severely ill due to this disease. A screening and enrollment log will be maintained.

There is no expectation of severe adverse outcomes or reactions due to a patient being treated with niclosamide. Niclosamide is generally well tolerated and has been prescribed clinically for over 30 years. Reported AEs in current use are mild and include nausea and diarrhea. All participants will be given access to contact information of the Study Team, and any adverse reactions will be reported as required by the protocol.

3 OBJECTIVES AND ENDPOINTS

The primary objective of this study is to determine if a course of treatment with niclosamide improves respiratory viral clearance compared to treatment with placebo.

Secondary objectives include comparing the viral shedding from fecal samples and severity of clinical outcomes between treatment groups.

Primary Efficacy Endpoint:

- Respiratory viral clearance at Day 3.

Secondary Efficacy Endpoints:

- Fecal viral clearance at Day 14.
- Reduction in viral shedding as measured by oropharyngeal swab on days, 3, 7, 10, 14
- Reduction in fecal viral shedding as measured by fecal PCR on days, 3, 7, 10, 14 and 21
- Progression to severe COVID, as a composite endpoint defined as O2 saturation <92% on room air in two consecutive measurements at least 2 hours apart OR requirement of hospitalization OR need for artificial ventilation OR death.
- Time to resolution of fever

Safety endpoint: incidence of AEs

4 STUDY DESIGN

4.1 OVERALL DESIGN

This is a double-blinded randomized controlled trial of 200 adult outpatients with mild to moderate symptoms of COVID-19 not requiring hospitalization. Participants will be identified as those reporting to

Tufts Medical Center seeking COVID-19 testing. All patients will be provided the option to participate in our study pending a positive SARS-CoV-2 test result. The Study Team will enroll and randomize patients into the study after the patient has a confirmed positive test result and meets all the inclusion and none of the exclusion criteria.

Participants in the treatment arm (n= 100) will receive niclosamide 2g orally daily for 7 days in addition to current standard of care treatment. Those in the control group (n=100) will receive placebo by mouth in the same numbers of pills on day 1 and daily for 6 more days (total 7 days of treatment) in addition to current standard of care treatment.

Laboratory PCR testing will be performed at Tufts Medical Center laboratory or other CLIA certified lab. Study participants will be recruited for participation from Tufts Medical Center. Associated sites at Newton-Wellesley Hospital, Melrose Wakefield and Lowell General Hospital as well as CRN sites will be included in recruitment and engaged as deemed necessary by the Study Team to ensure identification and reporting of maximum number of positive cases and prompt patient engagement/ enrollment.

A study recruitment brochure will be distributed at the associated Wellforce and CRN sites. A study poster will be available for affiliated outpatient sites that perform COVID testing. Both will include a QR code for ease of accessing study information. In addition, nurses who provide results to patients (by phone) who have tested positive at Tufts Medical Center outpatient will provide basic, scripted study information to potential participants. This information will include contact information for the Study Team and has been submitted to the Tufts IRB. Participation at Wellforce and CRN sites will be limited to distribution of study brochures and display of posters and (on a site by site basis) provision of PHI (name and contact phone number of potential participants who express interest in the study). This information will be provided to the Tufts Study Team via a secure, encrypted message. Revisions to the study brochure or any recruitment material will be provided to the site contact by the Tufts Study Team. No research activities will be conducted at the associated sites (e.g., informed consent, study visits, data collection, data entry, data analysis, etc.). All study activity will be conducted by the Tufts Study Team. The Tufts Study Team will keep record of the site of origin for each enrolled study participant. This de-identified, aggregated recruitment data will be provided to each site as requested by the site.

A study recruitment brochure will be distributed in the community. A study poster will be available for affiliated outpatient sites that perform COVID testing. Both will include a QR code for ease of accessing study information. In addition, nurses who provide results to patients (by phone) who have tested positive will provide basic, scripted study information to potential participants. This information will include contact information for the Study Team and has been submitted to the Tufts IRB.

Potential participants will initially be identified as those reporting to Tufts Medical Center, Wellforce or CRN outpatient. The Study Team will enroll and randomize patients into the study after the patient has a confirmed positive test result and meets all the inclusion and none of the exclusion criteria.

Participants who provide informed consent and meet all of the inclusion and none of the exclusion criteria will be randomized on Treatment Day 0 in a 1:1 ratio to either the Treatment Group or the

Control Group, in accordance with a computer-generated schedule prepared by a biostatistician. The randomization schedule will be incorporated into the REDCap Electronic Data Capture (EDC) system. Randomization will then be performed by study personnel directly in the EDC system. Study personnel will be instructed not to randomize until participant has been confirmed to meet all inclusion/exclusion criteria on treatment day 0.

4.2 SCIENTIFIC RATIONALE FOR STUDY DESIGN

Randomized double-blind placebo control (RDBPC) studies provide the strongest possible evidence of causation. Phase II trials are used to determine the efficacy and safety of an intervention in participants with the disease for which a new intervention is proposed. Randomization in combination with blinding helps to avoid possible bias in the selection of participants, their assignment to an intervention or control, and the analysis of their response to the intervention.

4.3 JUSTIFICATION FOR DOSE

The standard oral dosage of Niclosamide prescribed for Hymenolepis nana tapeworm infestation (e.g. adults—maximum of 2 grams/day for seven days) is clinically efficacious in this antiviral capacity. Based on previous antiviral studies of Niclosamide both in vitro and in vivo [5, 13, 14], the average required dosage to achieve antiviral activity is 1 uM, which corresponds to ~0.327 µg/ml. In a recent clinical trial to test efficacy of Niclosamide as an antimetastatic therapy [23, 24], clinicians found that upon oral intake, Niclosamide Cmax plasma level peaked to a median of 0.665 µg/ml (ranging from 0.429 to 0.848 µg/ml), suggesting that traditional oral drug delivery should be sufficient to inhibit SARS-CoV-2 production. Repeated dosing is commonly used when treating an infection where viruses may (at the time of ingestion) be at various stages of attachment, invasion, and replication.

Importantly, human infections with SARS-CoV-2 have demonstrated two known reservoirs of the virus. Viremia tends to be relatively uncommon, with viral replication largely occurring in the respiratory and gastrointestinal systems [37, 38]. The few cases in which SAR-CoV-2 titers are detectable in plasma have been reported in those patients already progressed to severe disease requiring hospitalization [39]. Plasma levels are limited both by oral absorption, but also by high plasma protein binding. In Bayer simulations Cmax levels reaching almost 1200 nmol/l, or 1.2 uM is predicted. There is 99% plasma protein binding. Though free drug concentrations may not exceed the IC50 in plasma, we do not anticipate viral burden in the plasma to be a significant contributor to disease or spread of virus.

While we firmly believe that standard oral delivery of Niclosamide will be sufficient to achieve anti-COVID-19 activity, it is important to address certain issues previously posed with regard to the pharmacokinetics of this drug. The inaugural publication describing studies of Niclosamide in human subjects reported that upon a single oral dose of 2g carbonyl-^{14}C-labeled Niclosamide, up to 25% of ^{14}C-activity was detected in urine while the remainder was eliminated in feces[31] suggesting that Niclosamide may be preferentially sequestered in the GI tract. Other studies have also suggested a similar biodistribution of Niclosamide in the colon[34]. Oral delivery of Niclosamide in mice infected with an epidemic strain of Clostridium difficile, inhibited disease pathogenesis by targeting host mechanisms

to prevent pathogen entry into intestinal epithelia without disrupting the endogenous gut microbiota[34]. With increasing insights emerging into the critical role of GI involvement in COVID-19 severity and transmission, this potential sequestration of Niclosamide in the gut could likely be beneficial for both treatment strategies as well as prevention of transmission.

The second major viral reservoir, the respiratory tract, may be a key site of activity for the niclosamide. Though lung tissue and epithelial lining fluid concentrations are not available, there is measurable activity in the respiratory tract. In a transgenic mouse model of asthma, niclosamide treatment (13 mg/kg/day) reduced mucus production, bronchoconstriction, and inflammation of airway tissues. Assuming the average human weighs approximately 62kg, our dosing strategy of 2g/day, is the equivalent of approximately 32mg/kg/day, which is significantly higher and likely to have similar effects. In addition, systemic delivery of niclosamide (20mg/kg) significantly enhanced efficacy of chemotherapeutic drugs in an in vivo mouse model of non-small cell lung cancer [40], further demonstrating that niclosamide can specifically target the lung in vivo.

4.4 END OF STUDY DEFINITION

A participant is considered to have completed the study if he or she has completed all phases of the study including the last visit or the last scheduled procedure shown in the ***Schedule of Activities, Section 1.2.***

Early Termination/Hospitalization

All participants have the right to withdraw from study participation at any time during the study. If, for whatever reason, a participant withdraws from the study or is hospitalized for increased severity of COVID symptoms or other cause, an ***Early Termination Visit*** will be performed as deemed feasible by the PI or physician Investigator.

Any AE, SAE or other medical condition or situation that occurs such that continued participation in the study would not be in the best interest of the participant will result in early termination.

The following procedures will be performed at the ***Early Termination Visit***:

- Assess for AEs
- Assess for complications following treatments
- Document all current medications, including medications over the counter and herbal medications
- Perform clinical assessment (as deemed feasible if hospitalized)
- Evaluate for increased severity of COVID-19-related disease

If clinical evaluation is deemed not feasible due to patient condition, the Study Team will collect as much of the ***Early Termination Visit*** data via the Electronic Medical Record as is available.

5 STUDY POPULATION

5.1 INCLUSION CRITERIA

In order to be eligible to participate in this study, an individual must meet all the following criteria:

- SARS-CoV-2 infection confirmed by PCR ≤ 3 days before randomization
- Provision of informed consent
- Stated willingness to comply with all study procedures and availability for the duration of the study
- Male or female, over 18 years of age
- No need for oxygen supplementation
- No requirement for hospitalization at the time of enrollment
- Ability to take oral medication and be willing to adhere to the Niclosamide/placebo regimen

5.2 EXCLUSION CRITERIA

An individual who meets any of the following criteria will be excluded from participation in this study:

- Known allergic reactions to components of the niclosamide
- Participation in another trial or use of any experimental treatment for COVID-19, including chloroquine, hydroxychloroquine, remdesivir, and lopinapir/ritonavir
- History of receipt of COVID-19 vaccine*
- Current hospitalization or requiring hospital admission at screening

* If a participant is enrolled in the study and then subsequently has an appointment for the COVID-19 vaccine, they will remain on study and will be advised that they can keep their vaccine appointment. Vaccine administration will be noted and considered in the final data analysis.

5.3 LIFESTYLE CONSIDERATIONS

No special preparations or additional steps (for example, special diets, fasting, other medicines, laxatives, or enemas) are necessary before, during, or immediately after taking niclosamide. Participants are advised to avoid alcohol consumption during the study treatment period.

5.4 SCREEN FAILURES

Screen failures are defined as participants who consent to participate in the clinical trial but are not subsequently randomly assigned to the study intervention or entered in the study. A minimal set of screen failure information is required to ensure transparent reporting of screen failure participants, to meet the Consolidated Standards of Reporting Trials (CONSORT) publishing requirements and to respond to queries from regulatory authorities. Minimal information includes demography, screen failure details, eligibility criteria, and any SAE.

5.5 STRATEGIES FOR RECRUITMENT AND RETENTION

Participants will be identified as those outpatients reporting to Tufts Medical Center, Wellforce or our CRN sites seeking COVID-19 testing. Any asymptomatic patient and those with mild to moderate COVID-19 symptoms not requiring hospital admission or supplemental oxygen, will be provided the option to participate in our study once they have a reported positive SARS-CoV-2 test result. Lowell General, St. Elizabeth's, Newton Wellesley Hospital, Maine Medical Center and New England Quality Care Alliance (NEQCA) have agreed to distribute our study brochure at the time of testing at their drive thru testing and ambulatory locations. These sites will also be displaying our study poster (with QR code) in the ED, Primary Care clinics. Additional Wellforce and CRN sites may be used to enhance recruitment. Study brochures and promotional material will be given to potential participants at each of the testing sites including study team contact information. The study brochure will be given to the patient at the time of COVID- 19 testing, by the testing site personnel. The participant will proactively contact the study team if interested in participation following a positive COVID-19 test.

The Study Team will enroll and randomize patients meeting the above criteria into the study after the patient has a confirmed positive test result and meets all of the inclusion and none of the exclusion criteria.

Tufts Community Health Improvement Program will incorporate the distribution of study material (brochure) with their general COVID-19 materials within the Chinatown community and residential towers. The Tufts Medical Center (TMC) Symptom Clinic, staffed by TC registered nurses (RNs), will be contacting patients who test positive by phone. The RN will give a brief (scripted) overview of the study and contact information for the study team at the time of call to provide testing result.

Social media will be used to enhance recruitment. This study will be listed at *www.clinicaltrials.gov*.

Participants will be offered twenty dollars ($20) for time and effort (via ClinCard) at each specimen collection timepoint (Days 1, 3, 7, 10, 14 and 21).

All research activity including Informed Consent will be performed by Tufts Medical Center employees.

6 STUDY INTERVENTION

6.1 STUDY INTERVENTION(S) ADMINISTRATION

6.1.1 STUDY INTERVENTION DESCRIPTION

Informed Consent will be obtained, all the inclusion and none of the exclusion criteria are met.

In addition to niclosamide or placebo treatments, all enrolled patients will be provided a home thermometer as well as a fingertip probe pulse oximeter, with the specific instructions to monitor both temperature at oxygen saturation at the time of daily oral administration of drug. The relatively low

reported percentage of error for the finger probe supports that the finger probe is the modality of choice to measure intermittent oxygen saturation in the outpatient setting.

Oropharyngeal and fecal swabs will be collected at days 1 (baseline), 3, 7, 10, and 14 of the study. An additional fecal specimen will be collected at day 21. In the case of oropharyngeal samples, care will be taken to ensure that sampling methods are consistent for each individual patient across all included timepoints, to limit any bias due to potential differences in viral load.

Oropharyngeal collection is minimally invasive and can reliably be self-administered. Recent research testing SARS-CoV-2 detection, nasopharyngeal and oropharyngeal (saliva) samples from confirmed SARS-CoV-2 found that oropharyngeal yielded greater detection sensitivity and consistency throughout the course of infection. Less variability in self-sample collection was also found. This research demonstrated that oropharyngeal samples are a viable and more sensitive alternative to nasopharyngeal swabs and can enable at-home self-administered sample collection for accurate SARS-CoV-2 testing [41, 42].

The collection of oropharyngeal samples will be directly observed by a Study Team member via the telehealth platform. The participant will open the viral transport kit and swab the back of the throat and tonsil area (avoiding mouth, teeth, and gums) and place to swab back into the vial. For fecal specimen, the participant will swab feces from a plastic container (or wrap) placed on the toilet seat. Printed instructions will be provided to each participant with detail on how to collect both the oropharyngeal and fecal specimens. These instructions will also be reviewed with the participant by a member of the Study Team.

Once obtained, samples will be transported by no contact pickup and delivery FedEx service to the Tufts Medical Center or CLIA- certified lab to prevent unnecessary hospital visits and to encourage compliance given the self-quarantine status of enrolled patients.

<u>Telemedicine Platform</u>

A HIPPA compliant telehealth platform; e.g., Doximity, will be used to conduct remote study visits.

Twice daily a list of COVID-19 positive patients will be generated by the Tufts Medical Center lab and provided to the Study Team via a secure Tufts email account. Upon receiving the COVID-19 positive test notification, COVID-19 positive participants will be approached remotely by a study Investigator for remote (telephone) Informed Consent. Once consent is obtained, all the
and none of the exclusion criteria are met, the study CRC will engage with the participant to enroll them in the telehealth platform and schedule the follow-up telehealth study appointments.

If the participant does not have a Smartphone, one will be provided to them along with the other study materials. A prepaid USPS envelope with additional instructions will be provided to send the package through the USPS for return. Participants will be guided thru the step-by-step telemedicine setup on their Smartphone by the study CRC. Scripted guidance will be provided to the study CRCs to ensure that

instructions provided to participants are consistent. For example, included in the script, the study CRC will provide instruction in simple, easy to understand language such as, "*I am going to take a minute and walk you through signing up for the app so you are ready on your visit date. "I've have actually gone ahead and scheduled the visit for you in the app". The app is navy blue, has a picture of a doctor with a stethoscope and lighter blue heart".* Once set up, a reminder of the upcoming appointment will be sent to the participant via email and text message.

Once the participant "checks- in" for their study visit, the CRC, investigator, or Study Team member will receive a notice that the participant is in the *Waiting Room*. A family member or other support person of the participant's choice can be included in any/all visits via Facetime. More than one study team member can also join the appointment as needed or desired with the *"Add Person"* Facetime number/address. Once the study team enters the *Waiting Room*, a split screen clearly displays the patient, support person and the study team member(s).

6.1.2 DOSING AND ADMINISTRATION

Participants eligible for the study will review and undergo informed consent at the time they receive confirmation of positive SARS-CoV-2 test result. After obtaining consent, participants will be randomly assigned on a 1:1 basis to receive:

- **Treatment Group blinded:** Will receive niclosamide 2g orally daily for 7 days in addition to current standard of care
- **Control Group blinded:** Will receive placebo by mouth daily for 7 days in addition to current standard of care

Niclosamide may be taken on an empty stomach (either 1 hour before or 2 hours after a meal). However, to prevent stomach upset, it is best taken after a light meal (for example, breakfast). Participants will be advised to avoid alcohol consumption during study treatment period.

6.2 PREPARATION/HANDLING/STORAGE/ACCOUNTABILITY

6.2.1 ACQUISITION AND ACCOUNTABILITY

In accordance with recent COVID-19 FDA Guidance, if scheduled visits at clinical sites are significantly impacted, certain IP, such as those that are typically distributed for self-administration, may be amenable to alternative secure delivery methods. As such:

Niclosamide and placebo (Investigational Product, IP) will be stored in the Tufts Medical Center Investigational Pharmacy. Upon enrollment, the investigational pharmacy will package and dispense the IP to the patient, along with the sampling kit. The entire supply of study medication will be dispensed at one time. It will be delivered to the subject via courier service. Subjects will be asked to document compliance with study protocol in a subject diary. If a subject does not use all the IP, the remainder shall be returned to the study investigators with the day 14 samples. The IP will then be returned to the manufacturer or destroyed on site.

Investigational new drug application (IND) submitted under section 505(i) of the Federal Food, Drug, and Cosmetic Act (FDCA) for Niclosamide chewable tablets, 500 mg has been approved: FDA IND # 151423.

Placebo manufacturer KABS Laboratories Inc. See Appendix A for manufacturing detail.

6.2.2 FORMULATION, APPEARANCE, PACKAGING, AND LABELING

Niclosamide will be provided as 500 mg chewable tablets. They are gray-yellow round tablets with FE printed on one side and the "Bayer Cross" on the other side. The excipients include: corn starch, talc, sodium lauryl sulphate, povidone, vanillin, magnesium stearate, saccharin sodium. They will be provided in blister packages for ease of use and enhanced stability. They will be labeled as "Niclosamide OR Placebo," with adequate administration instructions prior to dispensing to subjects.

6.2.3 PRODUCT STORAGE AND STABILITY

Niclosamide and the placebo are oral tablets. They can be stored at room temperature, 25°C (77°F) with excursions permitted to 15-30°C (59-86°F). They will be dispensed in blinded blister packaging.

6.2.4 PREPARATION

There will be no significant preparation. Both niclosamide and placebo tablets will be dispensed in blinded blister packages and labeled in accordance with state and federal regulation.

6.3 MEASURES TO MINIMIZE BIAS: RANDOMIZATION AND BLINDING

Participants who sign informed consent and meet all inclusion and exclusion criteria will be randomized on Treatment Day 0 in a 1:1 ratio to either the Treatment Group or the Control Group, in accordance with a computer-generated schedule prepared by a biostatistician. The randomization schedule will be incorporated into the REDCap EDC system. Randomization will then be performed by study personnel directly in the EDC system. Study personnel will be instructed not to randomize until participant has been confirmed to meet all inclusion/exclusion criteria on treatment day 0.

As all members of the Study Team will be blinded, Tufts Investigational Drug Services (IDS) will be unblinded and will dispense both the Niclosamide and placebo. The study intervention (niclosamide) and placebo will be packaged and as indistinguishable as possible.

Refer to ***Section 9, Statistical Considerations*** for sample size calculations.

6.4 STUDY INTERVENTION COMPLIANCE

During the *Baseline and Screening (Day 0),* the Principal Investigator (PI) or a co-Investigator will review the study with the participant and obtain informed consent and Health Insurance Portability and

Accountability Act of 1996 (HIPAA) authorization. A second member of Study Team (e.g., the study Clinical research coordinator (CRC)) will serve as a witness to the informed consent. After the informed consent is obtained, the participant will be assigned a unique enrollment number. The subject will then be randomized to treatment in EDC and provided blinded treatment (either Niclosamide or placebo) and other study materials via courier.

On ***Treatment Day*** *1*, a member of the Study Team or study CRC will review the dosing and schedule of Niclosamide/ placebo.

- The patient will be instructed to record all doses in Study Drug Administration Diary
- During each telehealth visit, the study CRC or other Study Team member will review the diary with the participant
- The participant will be advised not to discard any study pill bottles
- Baseline AEs, O2 saturation, temperature, oropharyngeal & fecal sample collection will be obtained

Refer to the ***Section 1.2, Schedule of Activities.***

6.5 CONCOMITANT THERAPY

Concomitant therapies are any new or existing medications or therapy taken by the patient including:

- Drugs, including but not limited to, prescription, over the counter, birth control pills/patches/hormonal devices, and homeopathic preparations
- Nondrug therapies, including but not limited to, thermal/laser/radiation procedures, vitamins, herbal medicines/supplements.

During the Screening process, information on all concomitant therapies, medications, and procedures will be recorded in the source documents and appropriate Case Report Form (CRF) along with the diagnosis or reason for use. Once the patient receives the first dose of study drug, recording of concomitant therapies will be limited to any new medication or modification of an existing medication taken for treatment of an AE. These therapies will be recorded in the source documents and appropriate CRF along with the diagnosis or reason for use. Those therapies used for the treatment of an adverse event are to be linked to an AE and documentation of the AE must also be completed

6.5.1 RESCUE MEDICINE

The study site will not supply rescue medication.

7 STUDY INTERVENTION DISCONTINUATION AND PARTICIPANT DISCONTINUATION/WITHDRAWAL.

7.1 DISCONTINUATION OF STUDY INTERVENTION

This study may be temporarily suspended or prematurely terminated if there is enough reasonable cause. Written notification, documenting the reason for study suspension or termination, will be

provided by the suspending or terminating party to the study team, the Sponsor and the Institutional Review Board (IRB), as appropriate. If the study is prematurely terminated or suspended, the study team will promptly inform the IRB and will provide the reason(s) for the termination or suspension.

Discontinuation from niclosamide does not mean discontinuation from the study, and remaining study procedures should be completed as indicated by the study protocol. If a clinically significant finding is identified (including, but not limited to changes from baseline) after enrollment, the investigator or qualified designee will determine if any change in participant management is needed. Any new clinically relevant finding will be reported as an AE.

7.2 PARTICIPANT DISCONTINUATION/WITHDRAWAL FROM THE STUDY

All participants are free to withdraw from participation at any time, for any reason, specified or unspecified, and without prejudice. Reasonable attempts will be made by the Investigator to provide a reason for participant withdrawals. The reason for the participant's withdrawal from the study will be specified in the participant's source documents and the CRF. The study team will make every effort to contact participants who are lost to follow-up. Attempts to contact such participants will be documented in the participant's records (e.g., times and dates of attempted telephone contact, receipt for sending a registered letter, etc.).

A participant will be discontinued from the study for the following reasons only:

- Participant withdrawal of consent
- Lost to follow-up
- Participant death
- Hospitalization for severe COVID- 19 symptoms

The following procedures will be performed at the ***Early Termination visit***:

- Assess for AEs
- Assess for complications following treatments
- Document all current medications, including medications over the counter and herbal medications
- Perform clinical assessment (as deemed feasible if hospitalized)
- Evaluate for increased severity of COVID-19-related disease

If clinical evaluation is deemed not feasible due to patient condition, the Study Team will collect as much ***Early Termination Visit*** data as possible from the Electronic Medical Record

The reason for participant discontinuation or withdrawal from the study will be recorded on the CRF.

7.3 LOST TO FOLLOW-UP

A participant will be considered lost to follow-up if he or she fails to complete more than (2) scheduled telehealth visits and is unable to be contacted by the study staff.

The following actions must be taken if a participant fails to attend a required telehealth visit:

- The Study Team will attempt to contact the participant and reschedule the missed visit and counsel the participant on the importance of maintaining the assigned visit schedule and ascertain if the participant wishes to and/or should continue in the study.
- Before a participant is deemed lost to follow-up, the investigator or designee will make every effort to regain contact with the participant (where possible, 3 telephone calls and, if necessary, a certified letter to the participant's last known mailing address or local equivalent methods). These contact attempts will be documented in the participant's medical record or study file.
- Should the participant continue to be unreachable, he or she will be considered to have withdrawn from the study with a primary reason of lost to follow-up.
- For participants considered lost to follow-up, the CRF will be completed up to the last contact with the participant.

8 STUDY ASSESSMENTS AND PROCEDURES

8.1 EFFICACY ASSESSMENTS

The specific timing of procedures/evaluations to be done at each study visit are found in ***Section 1.2, Schedule of Activities.*** All assessment will be performed by the study investigators or a qualified member of the study team.

<u>Baseline and Screening (Day 0)</u>

The following procedures will be performed at the Baseline/Screening visit:

- Review the study with the participant and obtain written informed consent and Health Insurance Portability and Accountability Act of 1996 (HIPAA) authorization
- Assign the participant a unique enrollment number
- Review and record medical history, surgical history, and medication history to determine eligibility based on inclusion/exclusion criteria
- Record smoking history
- Record demographics (age, race, ethnicity, gender)
- Document all current medications, including medications over the counter and herbal medications
- Record COVID symptoms, if symptomatic
- Record confirmation of positive SARS-CoV-2 test result
- Record number of days since onset of symptoms, if symptomatic

<u>Randomization and Treatment (Day 0)</u>

- Confirm patient meets all inclusion and none of the exclusion criteria
- Randomize subject to treatment in EDC

- Provide blinded treatment (either niclosamide or placebo) and other study materials via courier

Follow-Up Evaluation

Remote clinical follow-up will occur at the following time points: Days 1, 2, 3, 7, 10, 14, 21 and 30.

The following procedures will be performed at all follow-up visits, expected to be done by tele-medical methods, unless otherwise noted:

- Assess for AEs
- Assess COVID signs and symptoms
- Document all current medications, including medications over the counter and herbal medications
- Document patient's status as an outpatient, subsequently hospitalized, or died
- Perform clinical assessment by patient report and vital signs (temperature and oxygen saturation)
- Evaluate for increased severity of COVID-19-related disease
- Collect temperature and oximetry data daily, with the caveat that if O2 goes below 92%, an addition oximeter reading should be taken 2 hours later. If the follow-up O2 saturation remains below 92%, the participant will be referred to their Primary Care Physician (PCP) or to the local Emergency Department.
- Collect oropharyngeal swab samples for viral shedding on days 1, (baseline), 3, 7, 10, 14 (courier transport of specimen)
- Collect fecal samples for viral shedding as measured by fecal PCR on days 1, (baseline), 3, 7, 10, 14 and 21 (courier transport of specimen)

Final Study Visit (Non-hospitalized Participants)

The following procedures will be performed at the final post treatment visit:

- Assess for AEs
- Assess for complications following treatments
- Document all current medications, including medications over the counter and herbal medications
- Perform clinical assessment to evaluate for increased severity of COVID-19-related diseases

Early Termination/Hospitalization

All participants have the right to withdraw from study participation at any time during the study. If, for whatever reason, a participant withdraws from the study or is hospitalized, an ***Early Termination Visit*** will be performed.

The following procedures will be performed at the ***Early Termination Visit***:

- Assess for adverse events
- Assess for complications following treatments
- Document all current medications, including medications over the counter and herbal medications
- Perform clinical assessment (as deemed feasible if hospitalized)

- Evaluate for increased severity of COVID-19-related disease

If clinical evaluation is deemed not feasible due to patient condition, the Study Team will collect ***Early Termination Visit*** data via the Electronic Medical Record.

A 30-day follow-up call and AE assessment for those patients who are hospitalized will be performed.

8.2 SAFETY AND OTHER ASSESSMENTS

For Study procedures and evaluations to be done as part of the study to monitor safety and support the understanding of the study intervention's safety refer to ***Section 1.2, Schedule of Activities and Section 8.1 Study Assessments.***

8.3 ADVERSE EVENTS AND SERIOUS ADVERSE EVENTS

Adverse event data will be summarized for all participants in the safety population. Site-reported serious adverse events and unexpected adverse drug reactions will be summarized as participant-based counts and percentages by AE category. MedDRA system organ class and preferred term In addition, participant listings will be provided for serious, unexpected, and unanticipated adverse reactions. Any adverse events leading to discontinuation of study drug or death will also be listed for all participants.

Definition of Adverse Event (AE)
An AE is defined as any unanticipated medical occurrence regardless to relationship of the investigative arm of the trial. An AE can be any unintended sign, lab abnormality, symptom, or disease associated with the trial. Any abnormality that presents during a medical test are to be defined as an AE if it produces clinical signs and/or symptoms, requires intervention, or deemed clinically significant by the Investigator.

The *Common Terminology Criteria for Adverse Events (CTCAE) version 5.0 will* be utilized for AE reporting the following study- specific AEs:

- Cough
- Dyspnea
- Hypoxia
- Nausea
- Vomiting
- Abdominal pain
- Pruritus
- Loss of appetite
- Dizziness
- Skin rash

Definition of Serious Adverse Event (SAE)

An AE is considered serious if it results in any of the following:

- results in death;
- is life-threatening (places the subject at immediate risk of death from the event as it occurred);
- requires inpatient hospitalization or prolongation of existing hospitalization;
- results in a persistent or significant disability/incapacity;
- results in a congenital anomaly/birth defect; or
- any other adverse event that, based upon appropriate medical judgment, may jeopardize the subject's health and may require medical or surgical intervention to prevent one of the other outcomes listed in this definition

Definition of Unexpected Adverse Reaction (UAE)

An adverse reaction, the nature or severity of which is not consistent with the applicable product information (e.g. Product Information/Summary of Product Characteristics). This would include any SAE on health or safety, any life-threatening problem or death caused by, or associated with a drug; or any other unanticipated serious problem associated with a drug that relates to the rights, safety, or welfare of subjects.

8.3.1 CLASSIFICATION OF AN ADVERSE EVENT.

8.3.1.1 SEVERITY OF EVENT

All AEs will be assessed by the study clinician using the CTCAE V. 5.0

- Grade 1: Mild; asymptomatic or mild symptoms; clinical or diagnostic observations only; intervention not indicated.
- Grade 2: Moderate; minimal, local or noninvasive intervention indicated
- Grade 3: Severe or medically significant but not immediately life-threatening; hospitalization or prolongation of hospitalization indicated

8.3.1.2 RELATIONSHIP TO STUDY INTERVENTION

A Study Team physician will oversee the evaluation of patient reported severity of AEs using the following categories:

Relationship to Study Products

All AEs must have their relationship to study intervention assessed by the clinician who examines and evaluates the participant based on temporal relationship and his/her clinical judgment. The degree of certainty about causality will be graded using the categories below.

**Definitely:**	The relationship of the AE and the drug or the study procedure can be established.
**Probably:**	While a clear relationship to the drug or to the study procedure cannot be established, the AE is associated with an expected AE or there is no other

	medical condition or intervention, which could explain the occurrence of such an event.
**Possibly:**	There is no clear relationship between the AE and the drug or study procedure; however, one cannot conclude that there is no relationship.
**Unrelated:**	There is no relationship between the AE and the drug or study procedure. This may include but is not limited to the incident being an expected outcome of a previously existing or concurrent disease, concomitant medication or procedure the participant experienced.

8.3.1.3 EXPECTEDNESS

Study Team members who are clinically qualified (e.g., a physician co- Investigator) will be responsible for determining whether an AE is expected or unexpected. An AE will be considered unexpected if the nature, severity, or frequency of the event is not consistent with the risk information previously described for the study products. This information will be provided to the IRB, to the Study Sponsor, and to any relevant governmental agency with regulatory or public health authority.

8.3.2 TIME PERIOD AND FREQUENCY FOR EVENT ASSESSMENT AND FOLLOW-UP

The occurrence of an AE or SAE may come to the attention of study personnel during study visits and interviews of a study participant presenting for medical care.

All AEs including local and systemic reactions not meeting the criteria for SAEs will be captured on the appropriate CRF. Information to be collected includes event description, time of onset, clinician's assessment of severity, relationship to study product (assessed only by those with the training and authority to make a diagnosis), and time of resolution/stabilization of the event. All AEs occurring while on study must be documented appropriately regardless of relationship. All AEs will be followed to adequate resolution.

8.3.3 ADVERSE EVENT REPORTING

Adverse event data will be summarized for all participants in the safety population. Serious adverse events and unexpected adverse drug reactions will be summarized as participant-based counts and percentages by AE category. In addition, participant listings will be provided for serious, unexpected, and unanticipated adverse reactions. Any adverse events leading to discontinuation of study drug or death will also be listed for all participants.

8.3.4 SERIOUS ADVERSE EVENT REPORTING

Study team members who are qualified will immediately report any serious adverse event, whether or not considered study intervention related, including those listed in the protocol and must include an

assessment of whether there is a reasonable possibility that the study intervention caused the event. Study endpoints that are SAEs must be reported in accordance with the protocol.

8.3.5 REPORTING EVENTS TO PARTICIPANTS

AEs, SAEs and UPs will be reported to IRB as outlined in Sections 8.3.5, 8.3.6 and 8.4.2

8.3.6 EVENTS OF SPECIAL INTEREST

N/A

8.3.7 REPORTING OF PREGNANCY

During the Informed Consent discussion, the MD study Investigator will ask women of childbearing potential if they are pregnant. That yes/ no response will be recorded. We will not exclude women who are of childbearing years or pregnant. We will collect pregnancy status information at the time of enrollment. Niclosamide is a Category B (animal studies show no risks, but there are no controlled studies in pregnant women). Category B drugs include prenatal vitamins, acetaminophen and several other medications used routinely and safely during pregnancy.

In addition to recording whether women of childbearing age are pregnant at the time of study enrollment, we will also record their breastfeeding status. For enrolled subjects who are pregnant or breastfeeding, we will include follow up a specific query for any adverse effects on pregnancy and/or breastfeeding infant during the course of the study.

8.4 UNANTICIPATED PROBLEMS

8.4.1 DEFINITION OF UNANTICIPATED PROBLEMS (UP):

The Office for Human Research Protections (OHRP) considers unanticipated problems involving risks to participants or others to include, in general, any incident, experience, or outcome that meets **<u>all</u>** the following criteria:

- Unexpected in terms of nature, severity, or frequency given (a) the research procedures that are described in the protocol-related documents, such as the Institutional Review Board (IRB)-approved research protocol and informed consent document; and (b) the characteristics of the participant population being studied;
- Related or possibly related to participation in the research ("possibly related" means there is a reasonable possibility that the incident, experience, or outcome may have been caused by the procedures involved in the research); and
- Suggests that the research places participants or others at a greater risk of harm (including physical, psychological, economic, or social harm) than was previously known or recognized.

8.4.2 UNANTICIPATED PROBLEM REPORTING

The investigator will report unanticipated problems (UPs) to the reviewing Institutional Review Board (IRB). The UP report will include the following information:

- Protocol identifying information: protocol title and number, PI's name, and the IRB project number;
- A detailed description of the event, incident, experience, or outcome;
- An explanation of the basis for determining that the event, incident, experience, or outcome represents an UP;
- A description of any changes to the protocol or other corrective actions that have been taken or are proposed in response to the UP.

To satisfy the requirement for prompt reporting, UPs will be reported using the following timeline:

- UPs that are SAEs will be reported to the IRB within 5 working days of the investigator becoming aware of the event.
- A Study Team evaluation of an UP will be performed with a report of results of such evaluation will be provided to the reviewing IRB by the PI within 5 working days.
- All other Reportable New Information will be reported to the IRB as per the policy.

8.4.3 REPORTING UNANTICIPATED PROBLEMS TO PARTICIPANTS

AEs, SAEs and UPs will be reported to IRB as outlined in Sections 8.3.5, 8.3.6 and 8.4.2

9 STATISTICAL CONSIDERATIONS

9.1 STATISTICAL HYPOTHESES

The null hypothesis is that the proportion of participants with respiratory viral clearance at Day 3 is the same in both treatment groups. The alternative hypothesis is that they are different.

9.2 SAMPLE SIZE DETERMINATION

For the primary efficacy endpoint of respiratory virologic clearance at Day 3 measured by oropharyngeal viral shedding we assume that on Day 3, 50% of participants in the niclosamide group who have a positive result on Day 1 (prior to dosing) will have a negative test results and 15% in the placebo group. With 40 participants per group we have 89% power to show a statistically significant difference between groups at Day 3 using Fisher's Exact test at the two-sided 0.05 significance level. Assuming that 40% of randomized participants have a positive respiratory test result, 200 participants will be required to be randomized into the study.

9.3 POPULATIONS FOR ANALYSES

- Intention-to-Treat (ITT) Analysis Dataset (all randomized participants)

- Modified Intention-to-Treat Analysis Dataset (participants who took at least one dose of study intervention, have a positive test result on Day 1 and have Day 3 oropharyngeal sample results available for analysis.
- Safety Analysis Dataset: participants who took at least one dose of study intervention
- Per-Protocol Analysis Dataset: subset of the participants in the full analysis (ITT) set who took at least 80% of study intervention and had no protocol violations that would affect the primary efficacy endpoint.
- Other Datasets that may be used for sensitivity analyses

9.4 STATISTICAL ANALYSES

9.4.1 GENERAL APPROACH

Baseline demographic and clinical characteristics and other results will be summarized using descriptive summary statistics. Data collected in the trial will be summarized overall and by treatment arm. For continuous variables, results within each treatment arm will be summarized with the numbers of observations, mean, standard deviation, minimum, and maximum, as well as the 95% confidence interval for the mean. For treatment comparisons, the difference between the two treatment arms will be summarized with the difference of the two means and 95% confidence interval for the difference of the means. These calculations will be done under the assumption that the data for the two arms are independent and approximately normal in distribution. If not otherwise specified, the confidence interval for the difference of two means is calculated assuming unequal variance between the two groups. If asymptotic assumptions fail, nonparametric summary statistics (medians, 25th and 75th percentiles) may be displayed as an alternative. In addition, more appropriate non-parametric tests will be considered if the assumptions for the parametric tests are violated. For the comparison of two independent samples, if the data are not normally distributed, Wilcoxon rank-sum test will be performed instead of the parametric t-test.

For categorical variables, results within each arm will be summarized with participant counts, percentages, and 95% confidence intervals. The differences between the two treatment arms will be summarized with the difference in percentages and the asymptotic 95% confidence interval for the difference of two percentages.

Survival analysis techniques will be used to analyze the time-to-event variables. Survival curves will be constructed using Kaplan-Meier estimates. Log-rank test results will be computed for comparison of survival distributions. Summary tables for safety and efficacy endpoints will include event rates (Kaplan-Meier estimates of event rates), relative risk, confidence interval for the relative risk, the difference in means/rates, the confidence interval for difference in means/rates, and the p-value.

For the primary efficacy endpoint, respiratory Clearance at Day 3 will be summarized by treatment group and the difference will be tested using a two-sided Fisher's Exact Test at the 0.05 significance level. The corresponding confidence interval for the difference in proportions will be calculated.

Every effort will be made to ensure the oropharyngeal samples are collected at all time points specified by the protocol even if the subject discontinues the study drug. The primary analytic sample will be the mITT cohort

All analyses will be repeated using the per-protocol population.

9.4.2 ANALYSIS OF THE SECONDARY ENDPOINT(S)

Secondary Efficacy Analysis

- Fecal viral clearing at day 14 will be summarized by treatment group and the difference will be tested separately for each day using a two-sided Fisher's Exact Test.

- Respiratory viral shedding as measured on days 1, 3, 7, 10, 14 will be compared between treatment groups using repeated measures analysis.

- Fecal viral shedding as measured on days 1, 3, 7, 10, 14 and 21 will be compared between treatment groups using repeated measures analysis.

We plan to consider additional analysis approaches including but not limited to: time to negative COVID-19 test as determined by PCR using oropharyngeal and/or fecal samples; longitudinal analysis of continuous measures such as body temperature and O2 saturation; The proportion of participants in each treatment group who progress to severe COVID disease will be compared using a two-sided z test with continuity correction. The corresponding confidence interval for the difference in proportions will be calculated.

All secondary endpoint analyses will be performed for the mITT and PP populations at the 0.05 significance level. No adjustment for multiple testing will be performed.

9.4.3 SAFETY ANALYSES

Adverse event data will be summarized for all participants in the safety population. Site-reported serious adverse events and unexpected adverse drug reactions will be summarized as participant-based counts and percentages by AE category.

In addition, participant listings will be provided for serious, unexpected, and unanticipated adverse reactions. Any adverse events leading to discontinuation of study drug or death will also be listed for all participants.

9.4.4 BASELINE DESCRIPTIVE STATISTICS

Demographic characteristics and medical history, adverse events, COVID disease signs and symptoms and treatment compliance will be summarized and compared between treatment groups. The details of the statistical analyses will be included in the ***Statistical Analysis Plan.***

9.4.5 PLANNED INTERIM ANALYSES

N/A

9.4.6 SUB-GROUP ANALYSES

N/A

9.4.7 TABULATION OF INDIVIDUAL PARTICIPANT DATA

N/A

9.4.8 EXPLORATORY ANALYSES

A detailed description of all statistical analyses will be presented in the ***Statistical Analysis Plan.***

10 SUPPORTING DOCUMENTATION AND OPERATIONAL CONSIDERATIONS

10.1 REGULATORY, ETHICAL, AND STUDY OVERSIGHT CONSIDERATIONS.

10.1.1 INFORMED CONSENT PROCESS

10.1.1.1 CONSENT/ASSENT AND OTHER INFORMATIONAL DOCUMENTS PROVIDED TO PARTICIPANTS

Consent describing in detail the study intervention, study procedures, and risks will be provided to the participant and documentation of informed consent will be required prior to administering study interventions. Non-English speakers will be enrolled using interpreters and IRB approved Short Forms per the IRB's Short Form policy.

10.1.1.2 CONSENT PROCEDURES AND DOCUMENTATION

Informed consent (IC) is a process that is initiated prior to the individual's agreeing to participate in the study and continues throughout the individual's study participation. The PI or physician co-Investigator will explain the research study to the participant and answer any questions that may arise. Utilizing the telehealth platform, an explanation will be provided in terms suited to the participant's comprehension of the purposes, procedures, and potential risks of the study and of their rights as research participants. Potential participants will have the opportunity to ask questions. The participants will have the opportunity to discuss the study with their family or surrogates or think about it prior to agreeing to participate. They will be informed that participation is voluntary and that they may withdraw from the study at any time, without prejudice. The rights and welfare of the participants will be protected by emphasizing to them that the quality of their medical care will not be adversely affected if they decline to participate in this study.

FDA regulations generally require that the informed consent of a trial participant be documented by the use of a written consent form that has been approved by the IRB and signed and dated by the subject at the time of consent (21 CFR 50.27(a)). Considering COVID-19 infection control measures, if the technology is available, current FDA guidance suggests that electronic methods of obtaining informed consent should be considered as follows:

- To ensure that patients are approached in a consistent fashion, a standard process should be used that will accomplish the following:
  - Identification of who is on the call or telemedicine visit
  - Review of the IC with the patient by the investigator (or their designee) and response to any questions the patient may have
  - Confirmation by the witness that the patient’s questions have been answered
  - Confirmation by the investigator that the patient is willing to participate in the trial and sign the informed consent document while the witness is listening on the phone
  - Verbal confirmation by the patient that they would like to participate in the trial and that they have signed and dated the informed consent document that is in their possession.

- If the signed informed consent document cannot be collected from the patient’s location and included in the study records, FDA considers the following option acceptable to provide documentation that the patient signed the informed consent document:
  - A dated attestation by the witness who participated in the call and by the investigator that the patient confirmed that they agreed to participate in the study and signed the informed consent.

For this study involving participants with COVID-19 positivity, in accordance with FDA guidance, the following steps will be performed while obtaining the IC by phone or telehealth video chat from the Subject.

1) Purpose of the study and the potential risks/benefits of the use of the Investigational Agent Niclosamide in the treatment of confirmed COVID-19 infection will discussed in detail by the principal investigator or sub-investigator (PI/Sub-I) with the subject prior to obtaining the IC. Opportunity to review the ICF (informed consent form) prior to or during the discussion will be provided. Adequate time for discussion between the PI/Sub-I will be given to the potential participant. After review of the IC, any questions that the potential participant has will be addressed during or after review of the IC.

2) Availability and/or possibility of other potential treatment options will be discussed with the participant.

3) A second member of the study team will be present on the phone or video chat during the entire discussion. The witness will ask the potential participant if they understand the contents of the discussion and if they have any questions to address. The participant will be informed that they can ask questions at any time during the trial.

4) The PI/Sub-I will sign the ICF along with the witness. Copies of the signed form will be placed in the patient's medical record and provided to the participant.

5) This document will be placed into the electronic medical record and electronically signed, and time stamped by the investigator. In **de-identified form,** it will be the study source document for documenting the process of obtaining ICF. A copy of the electronically signed consent (signed by the witness and investigator) is either emailed, sent by US Mail to subject.

10.1.2 STUDY DISCONTINUATION AND CLOSURE

This study may be temporarily suspended or prematurely terminated if there is sufficient reasonable cause. Written notification, documenting the reason for study suspension or termination, will be provided by the suspending or terminating party to study participants, investigator, funding agency, and regulatory authorities. If the study is prematurely terminated or suspended, the Principal Investigator (PI) will promptly inform study participants and the Institutional Review Board (IRB) for the termination or suspension. Study participants will be contacted, as applicable, and be informed of changes to study visit schedule.

Circumstances that may warrant termination or suspension include, but are not limited to:

- Determination of unexpected, significant, or unacceptable risk to participants
- Insufficient compliance to protocol requirements
- Data that are not sufficiently complete and/or evaluable
- Determination that the primary endpoint has been met

The study may resume once concerns about safety, protocol compliance, and data quality are addressed and the IRB.

10.1.3 CONFIDENTIALITY AND PRIVACY

Participant confidentiality and privacy is strictly held in trust by the participating investigators, their staff, and the sponsor(s) and their interventions. This confidentiality is extended to cover testing of biological samples in addition to the clinical information relating to participants. Therefore, the study protocol, documentation, data, and all other information generated will be held in strict confidence. No information concerning the study, or the data will be released to any unauthorized third party without prior written approval of the sponsor.

All research activities will be conducted in as private a setting as possible. The use of the telehealth video platform will include participant instruction on using the platform in a private setting or with a family member/ significant other via Facetime as described in the Study Intervention.

Representatives of the Institutional Review Board (IRB), regulatory agencies or pharmaceutical company supplying study product may inspect all documents and records required to be maintained by the investigator, including but not limited to, medical and pharmacy records for the participants in this study. The clinical study site will permit access to such records.

The study participant's contact information will be securely stored at each clinical site for internal use during the study. At the end of the study, all records will continue to be kept in a secure location for as long a period as dictated by the reviewing IRB, Institutional policies, or sponsor requirements.

The Principal Investigator will be responsible to ensure the study is conducted in accordance with the protocol, Good Clinical Practice (GCP), applicable regulatory requirements, and that the data recorded is valid. To achieve this objective, the study will be continuously monitored, and the study conduct reviewed on a weekly basis by the Study Team.

Monitoring will be conducted to ensure that the rights and well-being of human participants are protected, that the reported trial data are accurate, complete, and verifiable, and that the conduct of the trial follows the currently approved protocol/amendment(s), with GCP, and with applicable regulatory requirement(s).

A Clinical Monitoring Plan will be created by the Study Team and will describe in detail the personnel who will conduct the monitoring, at what frequency monitoring will be done, at what level of detail monitoring will be performed, and the distribution of monitoring reports.

10.1.4 FUTURE USE OF DATA

The collection of personal patient information will be limited to the amount necessary to achieve the aims of the research, so that no unneeded sensitive information is being collected.

Data from the EDC will be exported into Excel or SAS file format (password protected), which will then be used for data analysis. Only de-identified, not including the participant's contact or identifying information data will be used for data analysis.

10.1.5 KEY ROLES AND STUDY GOVERNANCE

Provide the name and contact information of the Principal Investigator.

**Principal Investigator**
Harry Selker, MD

Institute for Clinical Research and Health Policy Studies<br>Tufts Clinical and Translational Science Institute (CTSI)<br>Tufts University<br>Tufts Medical Center
800 Washington Street, # 63, Boston, MA 02111
Phone: (617) 636-5009<br>Fax: (617) 636-8023
Email

10.1.6 SAFETY OVERSIGHT

An appropriately credentialled Independent Safety Monitor (ISM) without association to the trial or conflicts of related to the study will monitor the data on a weekly basis and make recommendations to the PI with respect to the:

- Safety of the trial participants including adverse events (AEs), serious adverse events (SAE's), and unexpected problems
- The Independent Safety Monitor will review and evaluate all AEs and SAEs in a blinded fashion, however, can be unblinded as needed.
- The ISM will have access to unblinded SAE rates for participants randomized to niclosamide and placebo. Rates of non-serious AEs will also be compared between the two study groups.

Subjects will be monitored for adverse events (AEs) and serious adverse events (SAEs). A greater than 3x incidence of treatment related SAEs in the niclosamide treatment group vs placebo will be added to the protocol as the stopping threshold

The PI is personally responsible for conducting and supervising the conduct of human subject's research by protecting the rights, safety, and welfare of subjects under the investigator's care. The PI also must ensure that all the research conducted is done so in an ethical manner and in accordance with all federal, state, and local laws and regulations, institutional policies/procedures, the study protocol/plan, and the requirements of the IRB. Oversight is defined as management by overseeing the performance or operation of a person or group, watchful care, superintendence, general supervision. Any person serving as a PI has voluntarily accepted these responsibilities and is expected to fully comply with these requirements. To provide PI oversight and to ensure that the rights, safety, and welfare of research subjects is protected the PI will, at a minimum, confirm:

- Any individual to whom a task is delegated is qualified by education, training, and experience to perform the task.
- There is adequate training for all staff participating in the conduct of the study
- The PI or another qualified individual associated with the study is available to study subjects to answer questions or provide care during the conduct of the research
- All research staff adhere to the research plan (i.e., inclusion/exclusion criteria, safety assessments, safety monitoring and reporting of unanticipated problems).

Expected Oversight Practices

- The PI will work closely with the study team to ensure oversight of the research study and the necessary documentation of such activities. A sub- Investigator can cover for the PI when he is unavailable or is on vacation.
- A Delegation of Authority Log will be completed prior to opening to accrual. This log will be maintained accurately during the life of the study.
- A Training Log will be completed prior to opening to accrual. This log will be maintained accurately during the life of the study.
- The PI or delegated physician Investigator will sign-off on all study-related documents (i.e., eligibility verification) prior to subjects beginning study treatment per protocol to ensure the safety of the study participants
- The PI or delegated physician Investigator will evaluate and ascribe attribution, sign-off on all AEs and SAEs.
- The PI and Study Team will establish the method in which they will consistently communicate. At a minimum, this is intended to be through a combination of electronic, audio, and face to face interactions. Details and specifics of these interactions will be established by the PI and Study Team prior to activation of each study.
- Communication regarding SAEs will be documented in real-time. If the PI is not available, the delegated physician Investigator should be notified.
- Regular meetings with the PI to discuss subject participation, including AEs and treatment, will be established to ensure adequate oversight. This is in addition to the immediate availability of the PI, or delegated physician Investigator, to address SAE, protocol interpretation, safety monitoring or other urgent clinical needs. Involvement of the treating sub-investigator is recommended but not required.
- Timely and accurate documentation of oversight is required and will be provided by the PI consistent with FDA1572. The nature of oversight documentation is through written and electronic means.
- The PI or physician Investigator will complete urgent documents including initial SAE review and sign-off within 24 hours of notification of the event.
- All non-urgent regulatory documents including IRB submission/revisions, AE attribution assignments, AE grading will be completed by the PI or physician Investigator within 48 hours.
- Additional study documents including but not limited to research notes, email correspondences, and study logs are to be reviewed at least on a weekly basis by the PI and included with research documentation.

10.1.7 CLINICAL MONITORING

Clinical monitoring will be conducted to ensure that the rights and well-being of trial participants are protected, that the reported trial data are accurate, complete, and verifiable, and that the conduct of the trial is in compliance with the currently approved protocol/amendment(s), with International

Conference on Harmonisation Good Clinical Practice (ICH GCP), and with applicable regulatory requirement(s).

Refer to ***Section 10.1.6 Safety Oversite for details.***

10.1.8 QUALITY ASSURANCE AND QUALITY CONTROL

Quality control (QC) procedures will be implemented beginning with the data entry system and data QC checks that will be run on the database will be generated. Any missing data or data anomalies will be communicated to study PI or designee for resolution.

Following written Standard Operating Procedures (SOPs), the PI and co- Investigators will verify that the clinical trial is conducted, and data are generated, and biological specimens are collected, documented (recorded), and reported in compliance with the protocol, International Conference on Harmonisation Good Clinical Practice (ICH GCP), and applicable regulatory requirements (e.g., Good Laboratory Practices (GLP), Good Manufacturing Practices (GMP)).

The investigational site will provide direct access to all source data/documents, and reports for the purpose of monitoring, auditing and inspection by local and regulatory authorities.

10.1.9 DATA HANDLING AND RECORD KEEPING.

10.1.9.1 DATA COLLECTION AND MANAGEMENT RESPONSIBILITIES

Data collection is the responsibility of the Study Staff under the supervision of the Investigator. The Investigator is responsible for ensuring the accuracy, completeness, legibility, and timeliness of the data reported.

All source documents will be completed in a neat, legible manner to ensure accurate interpretation of data.

Any hardcopies of study visit worksheets will be provided for use as source document worksheets for recording data for each participant enrolled in the study. Data recorded in the electronic case report form (eCRF) derived from study source documents will be consistent with the data recorded on the source documents.

Clinical data, AEs, concomitant medications, and any other data collected from participants will be entered into a REDCap database. The data system includes password protection and internal quality checks, such as automatic range checks, to identify data that appear inconsistent, incomplete, or inaccurate. Clinical data will be entered directly from the source documents.

10.1.9.2 STUDY RECORDS RETENTION

We will follow the IRB's *HRP-073 - SOP - Records Retention Timeframe – Investigators (0.03)*

10.1.10 PROTOCOL DEVIATIONS

A protocol deviation is any noncompliance with the clinical trial protocol, International Conference on Harmonisation Good Clinical Practice (ICH GCP), or Manual of Procedures (MOP) requirements. The noncompliance may be either on the part of the participant, the Investigator, or the Study Team. As a result of deviations, corrective actions are to be developed by the site and implemented promptly.

These practices are consistent with ICH GCP:

- 4.5 Compliance with Protocol, sections 4.5.1, 4.5.2, and 4.5.3
- 5.1 Quality Assurance and Quality Control, section 5.1.1
- 5.20 Noncompliance, sections 5.20.1, and 5.20.2.

Protocol deviations will be summarized by type of deviation and treatment group. Protocol deviation summaries will be participant-based.

10.1.11 PUBLICATION AND DATA SHARING POLICY

The preparation and submittal for publication of manuscripts containing the study results shall be in accordance with a process determined by mutual written agreement among the study Sponsor and participating institutions. The publication or presentation of any study results shall comply with all applicable privacy laws, including, but not limited to, the Health Insurance Portability and Accountability Act of 1996.

This study will comply with the NIH Data Sharing Policy and Policy on the Dissemination of NIH-Funded Clinical Trial Information and the Clinical Trials Registration and Results Information Submission rule. As such, this trial will be registered at ClinicalTrials.gov, and results information from this trial will be submitted to ClinicalTrials.gov. In addition, every attempt will be made to publish results in peer-reviewed journals.

10.1.12 CONFLICT OF INTEREST POLICY

The independence of this study from any actual or perceived influence, such as by the pharmaceutical industry, is critical. Therefore, any actual conflict of interest of persons who have a role in the design, conduct, analysis, publication, or any aspect of this trial will be disclosed and managed. Furthermore, persons who have a perceived conflict of interest will be required to have such conflicts managed in a way that is appropriate to their participation in the design and conduct of this trial. The study leadership in conjunction has established policies and procedures for all study group members to disclose all conflicts of interest and will establish a mechanism for the management of all reported dualities of interest.

10.2 ADDITIONAL CONSIDERATIONS

10.3 ABBREVIATIONS

AE	Adverse Event
ANCOVA	Analysis of Covariance
CFR	Code of Federal Regulations
CLIA	Clinical Laboratory Improvement Amendments
CMP	Clinical Monitoring Plan
COC	Certificate of Confidentiality
CONSORT	Consolidated Standards of Reporting Trials
CRF	Case Report Form
DCC	Data Coordinating Center
DHHS	Department of Health and Human Services
DSMB	Data Safety Monitoring Board
DRE	Disease-Related Event
EC	Ethics Committee
eCRF	Electronic Case Report Forms
FDA	Food and Drug Administration
FDAAA	Food and Drug Administration Amendments Act of 2007
FFR	Federal Financial Report
GCP	Good Clinical Practice
GLP	Good Laboratory Practices
GMP	Good Manufacturing Practices
GWAS	Genome-Wide Association Studies
HIPAA	Health Insurance Portability and Accountability Act
IB	Investigator's Brochure
ICH	International Conference on Harmonisation
ICMJE	International Committee of Medical Journal Editors
IDE	Investigational Device Exemption
IND	Investigational New Drug Application
IRB	Institutional Review Board
ISM	Independent Safety Monitor
ISO	International Organization for Standardization
ITT	Intention-To-Treat
LSMEANS	Least-squares Means
MedDRA	Medical Dictionary for Regulatory Activities
MOP	Manual of Procedures
MSDS	Material Safety Data Sheet
NCT	National Clinical Trial
NIH	National Institutes of Health
NIH IC	NIH Institute or Center
OHRP	Office for Human Research Protections
PI	Principal Investigator
QA	Quality Assurance
QC	Quality Control
SAE	Serious Adverse Event
SAP	Statistical Analysis Plan

SMC	Safety Monitoring Committee
SOA	Schedule of Activities
SOC	System Organ Class
SOP	Standard Operating Procedure
UP	Unanticipated Problem
US	United States

10.4 PROTOCOL AMENDMENT HISTORY

The table below is intended to capture changes of IRB-approved versions of the protocol, including a description of the change and rationale. A Summary of Changes table for the current amendment is located in the Protocol Title Page.

Version	Date	Description of Change	Brief Rationale

11 REFERENCES

1. Wu, Y., C. Guo, L. Tang, Z. Hong, J. Zhou, X. Dong, H. Yin, Q. Xiao, Y. Tang, X. Qu, L. Kuang, X. Fang, N. Mishra, J. Lu, H. Shan, G. Jiang, and X. Huang, *Prolonged presence of SARS-CoV-2 viral RNA in faecal samples.* Lancet Gastroenterol Hepatol, 2020. **5**(5): p. 434-435. 7158584

2. Jeon, S., M. Ko, J. Lee, I. Choi, S.Y. Byun, S. Park, D. Shum, and S. Kim, *Identification of antiviral drug candidates against SARS-CoV-2 from FDA-approved drugs.* bioRxiv, 2020: p. 2020.03.20.999730.

3. Jung, E., S. Nam, H. Oh, S. Jun, H.J. Ro, B. Kim, M. Kim, and Y.Y. Go, *Neutralization of Acidic Intracellular Vesicles by Niclosamide Inhibits Multiple Steps of the Dengue Virus Life Cycle In Vitro.* Sci Rep, 2019. **9**(1): p. 8682. 6582152

4. Cairns, D.M., D. Boorgu, M. Levin, and D.L. Kaplan, *Niclosamide rescues microcephaly in a humanized in vivo model of Zika infection using human induced neural stem cells.* Biol Open, 2018. **7**(1). 5829514

5. Chen, W., R.A. Mook, Jr., R.T. Premont, and J. Wang, *Niclosamide: Beyond an antihelminthic drug.* Cell Signal, 2018. **41**: p. 89-96. 5628105

6. Andersen, P.I., K. Krpina, A. Ianevski, N. Shtaida, E. Jo, J. Yang, S. Koit, T. Tenson, V. Hukkanen, M.W. Anthonsen, M. Bjoras, M. Evander, M.P. Windisch, E. Zusinaite, and D.E. Kainov, *Novel Antiviral Activities of Obatoclax, Emetine, Niclosamide, Brequinar, and Homoharringtonine.* Viruses, 2019. **11**(10). 6832696

7. Fan, X., J. Xu, M. Files, J.D. Cirillo, J.J. Endsley, J. Zhou, and M.A. Endsley, *Dual activity of niclosamide to suppress replication of integrated HIV-1 and Mycobacterium tuberculosis (Beijing).* Tuberculosis (Edinb), 2019. **116S**: p. S28-S33.

8. Jurgeit, A., R. McDowell, S. Moese, E. Meldrum, R. Schwendener, and U.F. Greber, *Niclosamide is a proton carrier and targets acidic endosomes with broad antiviral effects.* PLoS Pathog, 2012. **8**(10): p. e1002976. 3486884

9. Marrugal-Lorenzo, J.A., A. Serna-Gallego, J. Berastegui-Cabrera, J. Pachon, and J. Sanchez-Cespedes, *Repositioning salicylanilide anthelmintic drugs to treat adenovirus infections.* Sci Rep, 2019. **9**(1): p. 17. 6327057

10. Wang, Y.M., J.W. Lu, C.C. Lin, Y.F. Chin, T.Y. Wu, L.I. Lin, Z.Z. Lai, S.C. Kuo, and Y.J. Ho, *Antiviral activities of niclosamide and nitazoxanide against chikungunya virus entry and transmission.* Antiviral Res, 2016. **135**: p. 81-90.

11. Xu, J., P.Y. Shi, H. Li, and J. Zhou, *Broad Spectrum Antiviral Agent Niclosamide and Its Therapeutic Potential.* ACS Infect Dis, 2020.

12. Xu, M., E.M. Lee, Z. Wen, Y. Cheng, W.K. Huang, X. Qian, J. Tcw, J. Kouznetsova, S.C. Ogden, C. Hammack, F. Jacob, H.N. Nguyen, M. Itkin, C. Hanna, P. Shinn, C. Allen, S.G. Michael, A. Simeonov, W. Huang, K.M. Christian, A. Goate, K.J. Brennand, R. Huang, M. Xia, G.L. Ming, W. Zheng, H. Song, and H. Tang, *Identification of small-molecule inhibitors of Zika virus infection and induced neural cell death via a drug repurposing screen.* Nat Med, 2016. **22**(10): p. 1101-1107.

13. Wu, C.J., J.T. Jan, C.M. Chen, H.P. Hsieh, D.R. Hwang, H.W. Liu, C.Y. Liu, H.W. Huang, S.C. Chen, C.F. Hong, R.K. Lin, Y.S. Chao, and J.T. Hsu, *Inhibition of severe acute respiratory syndrome coronavirus replication by niclosamide.* Antimicrob Agents Chemother, 2004. **48**(7): p. 2693-6. 434198

14. Mazzon, M., A.M. Ortega-Prieto, D. Imrie, C. Luft, L. Hess, S. Czieso, J. Grove, J.K. Skelton, L. Farleigh, J.J. Bugert, E. Wright, N. Temperton, R. Angell, S. Oxenford, M. Jacobs, R. Ketteler, M. Dorner, and M. Marsh, *Identification of Broad-Spectrum Antiviral Compounds by Targeting Viral Entry.* Viruses, 2019. **11**(2). 6410080

15. Sekulovski, N., A.E. Whorton, T. Tanaka, Y. Hirota, M. Shi, J.A. MacLean, J.R.L. Mola, K. Groesch, P. Diaz-Sylvester, T. Wilson, and K. Hayashi, *Niclosamide suppresses macrophage induced inflammation in endometriosis.* Biol Reprod, 2020.

16. Thatikonda, S., V. Pooladanda, and C.A.-O.h.o.o. Godugu, *Repurposing an old drug for new use: Niclosamide in psoriasis-like skin inflammation.* (1097-4652 (Electronic)).

17. Hochmair, M., B. Rath, L. Klameth, E. Ulsperger, C. Weinlinger, A. Fazekas, A. Plangger, R. Zeillinger, and G. Hamilton, *Effects of salinomycin and niclosamide on small cell lung cancer and small cell lung cancer circulating tumor cell lines.* Invest New Drugs, 2019.

18. Chai, W.H., Y.R. Li, S.H. Lin, Y.H. Chao, C.H. Chen, P.C. Chan, and C.H. Lin, *Antihelminthic Niclosamide Induces Autophagy and Delayed Apoptosis in Human Non-small Lung Cancer Cells In Vitro and In Vivo.* Anticancer Res, 2020. **40**(3): p. 1405-1417.

19. Boyapally, R., G. Pulivendala, S. Bale, and C. Godugu, *Niclosamide alleviates pulmonary fibrosis in vitro and in vivo by attenuation of epithelial-to-mesenchymal transition, matrix proteins & Wnt/beta-catenin signaling: A drug repurposing study.* Life Sci, 2019. **220**: p. 8-20.

20. Cabrita, I., R. Benedetto, R. Schreiber, and K. Kunzelmann, *Niclosamide repurposed for the treatment of inflammatory airway disease.* JCI Insight, 2019. **4**(15). 6693830

21. Miner, K., K. Labitzke, B. Liu, P. Wang, K. Henckels, K. Gaida, R. Elliott, J.J. Chen, L. Liu, A. Leith, E. Trueblood, K. Hensley, X.Z. Xia, O. Homann, B. Bennett, M. Fiorino, J. Whoriskey, G. Yu, S. Escobar, M. Wong, T.L. Born, A. Budelsky, M. Comeau, D. Smith, J. Phillips, J.A. Johnston, J.G. McGivern, K. Weikl, D. Powers, K. Kunzelmann, D. Mohn, A. Hochheimer, and J.K. Sullivan, *Drug Repurposing: The Anthelmintics Niclosamide and Nitazoxanide Are Potent TMEM16A Antagonists That Fully Bronchodilate Airways.* (1663-9812 (Print)).

22. Burock, S., S. Daum, U. Keilholz, K. Neumann, W. Walther, and U. Stein, *Phase II trial to investigate the safety and efficacy of orally applied niclosamide in patients with metachronous or sychronous metastases of a colorectal cancer progressing after therapy: the NIKOLO trial.* BMC Cancer, 2018. **18**(1): p. 297. 5856000

23. Burock, S., S. Daum, H. Tr√∂ger, T.D. Kim, S. Kr√ºger, D.T. Rieke, S. Ochsenreither, K. Welter, P. Herrmann, A. Sleegers, W. Walther, U. Keilholz, and U. Stein, *Niclosamide a new chemotherapy agent? Pharmacokinetics of the potential anticancer drug in a patient cohort of the NIKOLO trial.* Journal of Clinical Oncology, 2018. **36**(15_suppl): p. e14536-e14536.

24. *A Study of Niclosamide Enemas in Subjects With Active Ulcerative Proctitis or Ulcerative Proctosigmoiditis*. 2018: Clinicaltrials.gov.

25. Guan, W.J., Z.Y. Ni, Y. Hu, W.H. Liang, C.Q. Ou, J.X. He, L. Liu, H. Shan, C.L. Lei, D.S.C. Hui, B. Du, L.J. Li, G. Zeng, K.Y. Yuen, R.C. Chen, C.L. Tang, T. Wang, P.Y. Chen, J. Xiang, S.Y. Li, J.L. Wang, Z.J. Liang, Y.X. Peng, L. Wei, Y. Liu, Y.H. Hu, P. Peng, J.M. Wang, J.Y. Liu, Z. Chen, G. Li, Z.J. Zheng, S.Q. Qiu, J. Luo, C.J. Ye, S.Y. Zhu, and N.S. Zhong, *Clinical Characteristics of Coronavirus Disease 2019 in China.* N Engl J Med, 2020. 7092819

26. *Preliminary Estimates of the Prevalence of Selected Underlying Health Conditions Among Patients with Coronavirus Disease 2019 — United States, February 12–March 28, 2020. .* MMWR Morb Mortal Wkly Rep, (69): p. 382-386.

27. Survey, N.H.I., *Early release of selected estimates based on data from the 2018 National Health Interview Survey.* US Department of Health and Human Services, CDC, 2020.

28. Jin, X., J.S. Lian, J.H. Hu, J. Gao, L. Zheng, Y.M. Zhang, S.R. Hao, H.Y. Jia, H. Cai, X.L. Zhang, G.D. Yu, K.J. Xu, X.Y. Wang, J.Q. Gu, S.Y. Zhang, C.Y. Ye, C.L. Jin, Y.F. Lu, X. Yu, X.P. Yu, J.R. Huang, K.L. Xu, Q. Ni, C.B. Yu, B. Zhu, Y.T. Li, J. Liu, H. Zhao, X. Zhang, L. Yu, Y.Z. Guo, J.W. Su, J.J. Tao, G.J. Lang, X.X. Wu, W.R. Wu, T.T. Qv, D.R. Xiang, P. Yi, D. Shi, Y. Chen, Y. Ren, Y.Q. Qiu, L.J. Li, J. Sheng, and Y. Yang, *Epidemiological, clinical and virological characteristics of 74 cases of coronavirus-infected disease 2019 (COVID-19) with gastrointestinal symptoms.* Gut, 2020. 7133387

29. Hamming, I., W. Timens, M.L. Bulthuis, A.T. Lely, G. Navis, and H. van Goor, *Tissue distribution of ACE2 protein, the functional receptor for SARS coronavirus. A first step in understanding SARS pathogenesis.* J Pathol, 2004. **203**(2): p. 631-7. 7167720

30. Chang, Y.-W., T.-K. Yeh, K.-T. Lin, W.-C. Chen, H.-T. Yao, S.-J. Lan, Y.-S. Wu, H.-P. Hsieh, C.-M. Chen, and C.-T. Chen, *Pharmacokinetics of Anti-SARS-CoV Agent Niclosamide and Its Analogs in Rats.* Journal of Food and Drug Analysis, 2006. **14**.

31. Zhang, J.L., H.F. Si, X.F. Shang, X.K. Zhang, B. Li, X.Z. Zhou, and J.Y. Zhang, *New life for an old drug: In vitro and in vivo effects of the anthelmintic drug niclosamide against Toxoplasma gondii RH strain.* Int J Parasitol Drugs Drug Resist, 2019. **9**: p. 27-34. 6312869

32. Domalaon, R., P.M. De Silva, A. Kumar, G.G. Zhanel, and F. Schweizer, *The Anthelmintic Drug Niclosamide Synergizes with Colistin and Reverses Colistin Resistance in Gram-Negative Bacilli.* Antimicrob Agents Chemother, 2019. **63**(4). 6437516

33. Tam, J., T. Hamza, B. Ma, K. Chen, G.L. Beilhartz, J. Ravel, H. Feng, and R.A. Melnyk, *Host-targeted niclosamide inhibits C. difficile virulence and prevents disease in mice without disrupting the gut microbiota.* Nat Commun, 2018. **9**(1): p. 5233. 6286312

34. Farkouh, M.E., M. Domanski, G.D. Dangas, L.C. Godoy, M.J. Mack, F.S. Siami, T.H. Hamza, B. Shah, G.G. Stefanini, M.S. Sidhu, J.F. Tanguay, K. Ramanathan, S.K. Sharma, J. French, W. Hueb, D.J. Cohen, and V. Fuster, *Long-Term Survival Following Multivessel Revascularization in Patients With Diabetes: The FREEDOM Follow-On Study.* J Am Coll Cardiol, 2019. **73**(6): p. 629-638. 6839829

35. Hatamipour, M., M.R. Jaafari, A.A. Momtazi-Borojeni, M. Ramezani, and A. Sahebkar, *Development, characterization and evaluation of in vivo anti-tumor activity of niclosamide nanoliposomes against colon carcinoma.* Curr Mol Pharmacol, 2019.

36. Hatamipour, M., M.R. Jaafari, A.A. Momtazi-Borojeni, M. Ramezani, and A. Sahebkar, *Nanoliposomal Encapsulation Enhances In Vivo Anti-Tumor Activity of Niclosamide against Melanoma.* Anticancer Agents Med Chem, 2019. **19**(13): p. 1618-1626.

37. Huang C, wang Y, Li X, et al. (2020) Clinical features of patients infected with 2019 novel coronavirus in Wuhan, China. Lancet 395: 497-506.

38. Ding S, Lian TJ. (2020) Is SARS-CoV-2 Also an Enteric Pathogen With Potential Fecal– Oral Transmission? A COVID-19 Virological and Clinical Review. Gastroenterology. 5: 1-9.

39. Lescure, Francois-Xavier et al. (2020) Clinical and virological data of the first cases of COVID-19 in Europe: a case series. The Lancet Infectious Diseases. 20(6):697 – 706.

40. Luo, F., Luo, M., Rong, Q. et al. Niclosamide, an antihelmintic drug, enhances efficacy of PD-1/PD-L1 immune checkpoint blockade in non-small cell lung cancer. j. immunotherapy cancer 7, 245 (2019). https://doi.org/10.1186/s40425-019-0733-7.

41. Azzi L, Carcano G, Dalla Gasperina D, Sessa F, Maurino V, Baj A. Two cases of COVID-19 with positive salivary and negative pharyngeal or respiratory swabs at hospital discharge: A rising concern [published online ahead of print, 2020 Apr 25]. *Oral Dis*. 2020;10.1111/odi.13368. doi:10.1111/odi.13368

42. Wyllie, A.L., Fournier, J., Casanovas-Massana, A., Campbell, M., Tokuyama, M., Vijayakumar, P., Geng, B., Muenker, M.C., Moore, A.J., Vogels, C.B. and Petrone, M.E., 2020. Saliva is more sensitive for SARS-CoV-2 detection in COVID-19 patients than nasopharyngeal swabs. *Medrxiv*.

Statistical analysis plan -- Niclosamide for Patients with Mild to Moderate Disease from Novel Coronavirus (COVID-19)

Clinicaltrials.gov NCT04399356

SAP version 1, July 13, 2021, based on protocol version 8.0, February 2021

SAP contributors

Ludovic Trinquart

Lori Lyn Price

Background and rationale

Therapeutic approaches are needed to improve outcomes in patients with COVID-19. Niclosamide is an oral anthelminthic drug primarily used to treat parasitic infections. However niclosamide may have broad clinical applications to treat diseases other than those caused by parasites. Niclosamide has potent antiviral activity against single-stranded RNA viruses including coronaviruses. It was proposed as an antiviral during the SARS outbreak in 2002. It was found to inhibit SARS coronavirus, SARS-CoV, in *in vitro* studies and similarly structured RNA viruses (both *in vitro* and *in vivo)*. Niclosamide has antiviral properties for similarly structured pathogenic viruses, including Zika virus, adenovirus, dengue, and chikungunya virus.

Because the drug is inexpensive and has few if any side effects, taking Niclosamide prophylactically might help to prevent COVID-19 spreading. Even if this treatment does not completely eradicate infection, niclosamide treatment may help to decrease viral load, thereby allowing the host immune system to better combat the disease.

Objectives

Primary Objective: To evaluate the efficacy of niclosamide in shortening contagious period as determined by time to viral clearance.

Secondary Objectives: To evaluate the efficacy of niclosamide on symptoms and progression to severe COVID-19.

Study Methods

Trial design

The trial is a single-center, randomized, parallel-group, placebo-controlled trial. Treatment allocation was a 1:1 ratio. Patients were randomized to either niclosamide (2 grams orally once daily for 7 days) or matched placebo control.

Randomization

The random allocation sequence was computer-generated by a biostatistician. Randomization was stratified into three strata: Tufts lab and non-Tufts lab. We used blocking (block size 4). The random allocation sequence was implemented into the Redcap Electronic Data Capture (EDC) system which guaranteed concealment of the sequence until treatments were assigned.

Sample size

For the primary efficacy endpoint of respiratory virologic clearance at Day 3 measured by oropharyngeal viral shedding, 40 participants in each group achieve 89.1% power to detect a difference between the group proportions of 35%. We assumed that 50% and 15% of participants in the niclosamide and placebo groups would have a negative test on Day 3. The calculation was under a two-sided Fisher's Exact Test and a significance level of 0.05.

Framework

For all objectives, we test for superiority of niclosamide against placebo.

Statistical interim analyses and stopping guidance

We did not plan or perform any interim analyses.

The trial was closed to recruitment on June 22, 2021 due to the effective ending of the COVID-19 epidemic in Massachusetts resulting in lack of available candidates.

Timing of final analysis
Final analysis will take place in one stage, when every patient has reached 30-day follow-up and data for the primary and secondary endpoints have been received and cleaned.

Timing of outcome assessments, including visit windows
Primary efficacy endpoint: respiratory viral clearance at Day 3.

Secondary efficacy endpoints

• Fecal viral clearance at Day 14.
• Reduction in viral shedding as measured by oropharyngeal swab on days, 3, 7, 10, 14.
• Reduction in fecal viral shedding as measured by fecal PCR on days, 3, 7, 10, 14 and 21.
• Progression to severe COVID.
• Resolution of symptoms (including but not limited to fever, cough, fatigue).

Statistical principles

Confidence intervals and P values
All statistical tests will be 2-sided and will be performed using a 5% significance level. We will derive 95% two-sided confidence intervals.

Adherence and protocol deviations
In this study, four pills are to be taken daily for 7 days. Compliance is assessed by the percentage of subjects who have taken the scheduled number of pills:
% compliance = 100x (number of pills taken / 28 pills supposed to have been taken).
We will summarize compliance by randomization group: mean % compliance as well as number and percentage of participants with more than 80% compliance (24 pills out of 28).
Similarly, we will assess compliance with oropharyngeal and fecal sampling.

Protocol deviations will be classified into:

• PCR > 3 days before randomization
• taking the first dose before the first telehealth visit
• missing at least 1 telehealth visit
• collecting at least one oropharyngeal sample outside of window (day 1, 3, 7, 10, 14 ±1 day)
• collecting at least one fecal sample outside of window (day, 1, 3, 7, 10, 14, 21 ±1 day)
• collecting less than 5 oropharyngeal samples
• collecting less than 6 fecal samples
• taking the wrong dose at least once
• taking the wrong dose at least once
• missing a dose at least once
• expected assessments not completed during at least one telehealth visit (e.g., physician disconnected)

Protocol deviations were classified prior to unblinding of treatment assignment. We will report detailed protocol deviations per participant. We will summarize the number and percentage of participants with protocol deviations by treatment group with details of type of deviation.

Analysis populations
We will exclude participants who withdrew consent after randomization but before taking any sample or pill.

The intention-to-treat (ITT) population will include all randomized patients according to the treatment they were randomized to receive.

The per-protocol population will a subset of the participants in the full analysis (ITT) set who took at least 80% of study intervention.

The modified ITT population will include participants who took at least one pill, have a positive oropharyngeal test result on Day 1 and have Day 3 oropharyngeal sample results available for analysis.

The safety population will include participants who took at least one pill.

Trial population

Screening, eligibility, recruitment, and withdrawal/follow-up

We will use a CONSORT flow diagram to summarize the number of participants who were:

• assessed for eligibility at screening
• eligible at screening
• eligible and randomized
• withdrew prior to received first dose
• randomized and included in the primary analysis

The flow diagram will also show the numbers who were eligible but not randomized, who did not receive the randomized allocation, who were lost to follow-up, who discontinued the intervention, and we will describe the reasons.

Baseline patient characteristics

Participants will be described at the time of randomization with respect to age, sex, race/ethnicity, COVID-19 symptoms, smoking, obesity/overweight, asthma, COPD, cancer, cerebrovascular disease, CKD, cystic fibrosis, heart conditions, hypertension, immunocompromised state, liver disease, neurologic conditions, pulmonary fibrosis, sickle cell disease, thalassemia, diabetes, both overall and separately for the two randomization groups. We will summarize categorical data by numbers and percentages. We will summarize continuous data by mean, SD (or median, Q1-Q3 if data are skewed). We will not undertake tests of statistical significance. We will note the clinical importance of any imbalance.

Analysis

Outcome definitions

<u>Viral clearance</u>

Respiratory viral clearance is defined as the first day a participant's oropharyngeal sample result is negative, provided that none of the subsequent oropharyngeal sample results are positive. In primary analyses, we will use sample results (positive or negative) as returned by Diagnostic Solutions Laboratory. We will use a similar definition for fecal viral clearance. We will calculate the time to clearance since Day 1.

If a participant has several samples on the same day, we will consider the result to be positive if at least one of the sample results is positive, and negative otherwise.

In table 1, we illustrate possible patterns of time to viral clearance and time post-viral clearance. We illustrate samples available on days per protocol. But, according to our definition of clearance, time to clearance and time post-clearance are identifiable even if samples taken on different days.

Table 1: Patterns of sample results and time to viral clearance

Day 1	Day 3	Day 7	Day 10	Day 14	Clearance vs. censored	Time to clearance	Time post-clearance

+	+	+	-	-	1	10	4
-	-	-	-	-	1	1	13
-	+	-	+	+	0	14	0
-	+	-	-	-	1	7	7

In absence of deviations from protocol, we expect oropharyngeal sample results on 5 distinct days (day 1, 3, 7, 10, 14) and fecal sample on 6 distinct days (day 1, 3, 7, 10, 14, 21). Some participants may have samples on less than 5 or 6 distinct days. We will identify the occurrence of viral clearance as defined above based on available samples. If clearance is not observed, we will censor participants at their last available sample. In Table 2, we illustrate possible patterns of non-available sample results.

Table 2: Patterns of days without sample result available and time to viral clearance

Day 1	Day 3	Day 7	Day 10	Day 14	Clearance vs. censored	Time to clearance	Time post-clearance
+	+	NA	-	-	1	10	4
-	-	-	NA	NA	1	1	6
-	+	-	+	NA	0	10	0
-	+	NA	-	-	1	10	4

NA: not available.

Analysis methods

<u>Primary analysis</u>

The primary endpoint is viral clearance in respiratory samples at day 3. The primary analysis will be based on the ITT population (i.e., all randomized participants). We will estimate the cumulative probability of being in clearance in each randomization group by using the Kaplan-Meier estimator. We will compare the cumulative probability of clearance at day 3 between the two groups by using a chi-square test based on a log(− log(·)) transformation for the survival function.[Klein et al. Statist. Med. 2007; 26:4505-4519].

$$\chi^2 = \frac{\left\{\log\left(-\log\left(\hat{S}_1(t)\right)\right) - \log\left(-\log\left(\hat{S}_0(t)\right)\right)\right\}^2}{\frac{\hat{\sigma}_1(t)^2}{\log\left(\hat{S}_1(t)\right)^2} + \frac{\hat{\sigma}_0(t)^2}{\log\left(\hat{S}_0(t)\right)^2}}$$

Under the null hypothesis of no difference in probability of being in clearance between the two groups, the statistic of test is asymptotically χ_1^2-distributed. We will provide the between-group difference in probability of viral clearance at day 3 and the associated 95% confidence interval based on the cloglog transformation of the survival function.

<u>Secondary analyses</u>

In secondary analyses,

1) We will compare the respiratory clearance probability functions from day 1 to 14 between the two groups by using a log-rank test.
2) We will calculate the area under each respiratory clearance-free probability curve which gives the mean time to viral clearance up to day 14. We will estimate the mean difference in time post-viral clearance between groups and the associated 95% confidence interval.
3) We will compare how the proportion of negative oropharyngeal sample results evolved over time between treatment groups by using random-intercepts logistic regression models for longitudinal binary outcome data.
4) We will repeat the primary analysis for fecal viral clearance at day 14.
5) We will repeat the secondary analyses 1)-3) above for fecal viral clearance.
6) We will repeat the primary and secondary analysis by considering the combined oropharyngeal and/or fecal sample results. If both types of samples are available on a given day, we will consider the result to be positive if at least one of the sample results is positive, and negative otherwise. If only one type of sample is available on a given day and it is positive (respectively negative), the result will be positive (negative) for that day.
7) We will compare the proportion of participants who progressed to severe COVID disease between groups.
8) We will compare the proportions of participants free of symptoms. We will include participants who reported symptoms on Day 1 and we will assess the time to symptom resolution (symptom no longer reported). We will analyze 8 symptom categories described in Table 3.
9) We will repeat the primary and secondary analyses for viral clearance in the mITT population.
10) We will repeat the primary and secondary analyses for viral clearance in the per protocol population.

<u>Subgroup analyses</u>

We pre-specified subgroup analyses of viral clearance and symptom resolution according to BMI (<25 kg/m^2 vs. ≥25 kg/m^2) and according to diabetes.

<u>Exploratory analyses</u>

In exploratory analyses, we will repeat the primary and secondary analyses of viral clearance by calculating the time to viral clearance from the confirmatory PCR before randomization.

Moreover, in an analysis blinded to random allocation, we will analyze the trajectories of oropharyngeal and fecal sample results. In particular, we will identify participants with a negative test result followed by at least one positive test result (as defined by the DSL COVID-19 Assay); we will examine the Ct values of the positive test results following a negative test result. We will repeat the primary and secondary analyses by considering alternative thresholds to define a positive result, based on the blinded review of data.

We also will perform longitudinal analyses to assess the between-group difference in gene expression over time. For each participant and each sample, we will calculate the difference in expression (in terms of quantification cycle) between the target gene (SARS-CoV-2 specific nucleocapsid N1; panspecific CoV nucleocapsid N3; or SARS-CoV-2 spike) and a reference gene (RNase P): ΔCq= Cq(target gene)−Cq(reference gene). We will create spaghetti plots in each randomization group with lowess smoother curve superimposed. We will create heatmaps of ΔCq values scaled to the mean and standard deviation within each participant. We will use linear mixed models, including a term for the group, a term for time, and group x time interaction term and a subject-level random intercept, to compare the trajectories in normalized expression between groups over time.

Data will include a number of non-detects, i.e. reactions lacking a Cq value. Missing Cq value can correspond to a true Cq value above the Cq threshold. Alternatively it can correspond to zero expression (no amplification above the Cq threshold). Or it can correspond to a failure to detect a true Cq value below the Cq threshold. We will account for non-detects by setting undetermined Cq values at 40. In sensitivity analyses, we will account for non-detects by using hot deck imputation.

We will also consider the following modified ITT analyses of viral clearance:

- for the analysis based on oropharyngeal samples, fecal samples, and both types, we will include participants who took at least one pill, have a positive test result at any time during the trial and have Day 3 sample results available for analysis.

- for the analysis based on fecal samples, we will include participants who took at least one pill, have a positive oropharyngeal test result and have Day 3 fecal sample results available for analysis.

Missing data
For the longitudinal analyses, we will perform sensitivity analyses by imputing data according to sequential multiple imputation

Harms
The number (and percentage) of patients experiencing each adverse event will be presented, both for the overall safety population and in each randomization group. We will not perform statistical testing. We will assess the clinical significance of the differences. Adverse events will include abdominal pain, congestion or runny nose, cough, diarrhea, dizziness, dyspnea, fatigue, fever or chills, headaches, hypoxia, loss of appetite, muscle or body aches, nausea, new loss of taste or smell, pruritus, shortness of breath/difficulty breathing, skin rash, sore throat, vomiting, and other.

Statistical software
The analysis will be carried out by using SAS and R.

Table 3: Classification of symptoms into 8 categories

1.Central nervous system
Dizziness<br>Fatigue<br>Headache
2.Upper gastrointestinal
Appetite change<br>Nausea<br>Vomiting
3.Lower gastrointestinal
Abdominal pain<br>Diarrhea
4.Ear, nose, throat
Sore throat<br>Congestion<br>Loss of taste/smell
5.Respiratory/Pulmonary
Cough<br>Dyspnea/Shortness of breath<br>Hypoxia
6.Musculoskeletal
Muscle aches
Dermatologic
Rash<br>Pruritus
7.Systemic
Fever/Chills
8.Other
