# Supplementary material for: Efficacy of Niclosamide vs Placebo in SARS-CoV-2 Respiratory Viral Clearance, Viral Shedding, and Duration of Symptoms Among Patients With Mild to Moderate COVID-19: A Phase 2 Randomized Clinical Trial
Source: JAMA Netw Open. 2022 Feb 9;5(2):e2144942. doi: 10.1001/jamanetworkopen.2021.44942 (PMC8829666; doi:10.1001/jamanetworkopen.2021.44942)
Supplement: Supplement 3. — Data Sharing Statement [file jamanetwopen-e2144942-s003.pdf]

Data Sharing Statement

Cairns DM, Dulko D, Griffiths JK, et al. Efficacy of niclosamide vs placebo in SARS-CoV-2 respiratory viral clearance, viral shedding, and duration of symptoms among patients with mild to moderate COVID-19: a phase 2 randomized clinical trial. *JAMA Newtork Open*. 2022;5(1):e2144942. doi:10.1001/jamanetworkopen.2021.44942

Data
Data available: Yes
Data types: Deidentified participant data
How to access data: hselker@tuftsmedicalcenter.org
When available: With publication

Supporting Documents
Document types: Statistical/analytic code, Informed consent form
How to access documents: hselker@tuftsmedicalcenter.org
When available: With publication

Additional Information
Who can access the data: Anyone requesting the data shall be provided access.
Types of analyses: Data can be provided for any purpose.
Mechanisms of data availability: The data will be made available upon request.

© 2022 Cairns DM et al. *JAMA Network Open*.
